# Expanding the Topological Landscape by a G‐Column Flip of a Parallel G‐Quadruplex

**DOI:** 10.1002/chem.202101181

**Published:** 2021-06-04

**Authors:** Swantje Mohr, Jagannath Jana, Yoanes Maria Vianney, Klaus Weisz

**Affiliations:** ^1^ Institute of Biochemistry Universität Greifswald Felix-Hausdorff-Str. 4 17487 Greifswald Germany

**Keywords:** DNA, G-quadruplexes, NMR spectroscopy, quadruplex-duplex junction, topology

## Abstract

Canonical G‐quadruplexes can adopt a variety of different topologies depending on the arrangement of propeller, lateral, or diagonal loops connecting the four G‐columns. A novel intramolecular G‐quadruplex structure is derived through inversion of the last G‐tract of a three‐layered parallel fold, associated with the transition of a single propeller into a lateral loop. The resulting (3+1) hybrid fold features three *syn⋅anti⋅anti⋅anti* G‐tetrads with a 3’‐terminal all‐*syn* G‐column. Although the ability of forming a duplex stem‐loop between G‐tracts seems beneficial for a propeller‐to‐lateral loop rearrangement, unmodified G‐rich sequences resist folding into the new (3+1) topology. However, refolding can be driven by the incorporation of *syn*‐favoring guanosine analogues into positions of the fourth G‐stretch. The presented hybrid‐type G‐quadruplex structure as determined by NMR spectroscopy may provide for an additional scaffold in quadruplex‐based technologies.

## Graphical abstract

**Starting with a parallel‐stranded** DNA G‐quadruplex, refolding into a novel (3+1) hybrid topology with a propeller‐propeller‐lateral loop architecture and an all‐*syn* 3’‐terminal G‐tract is reported. Although supported by additional stacking of a lateral duplex hairpin onto an outer G‐tetrad, the structural rearrangement must be enforced through the specific incorporation of *syn*‐favoring 8‐bromoguanosine analogues into the terminal G‐run.

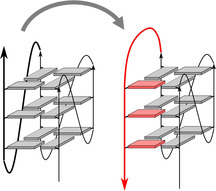

## Introduction

G‐quadruplexes (G4s) have evoked great interest in the last two decades due to their detection under *in vivo* conditions and their putative regulatory roles in gene expression, making them promising targets for novel therapeutic strategies.[^1^, ^2^] In addition, these structures have been increasingly employed as versatile tools in bio‐ and nanotechnological applications, e. g., in sensor systems, electronic switches, or as DNAzymes.[^3^, ^4^] In contrast to a B‐type genomic DNA duplex, quadruplex functions and properties heavily rely on their tetra‐stranded scaffold and may be exquisitely modulated by the remarkable G4 structural diversity. The latter depends on the nucleic acid sequence but also on the outer environment and determines G4 specific interactions with other molecules or ions.

G‐quadruplexes can be formed by the intramolecular folding of a single‐stranded nucleic acid comprised of four G‐runs interrupted by intervening nucleotides. Association of G residues from each of the four G‐tracts will form a quadruplex core with stacked G‐tetrads of four guanine bases held together in a square‐planar arrangement through cyclic hydrogen bonds. The stacked architecture is further stabilized by the coordination of metal ions like K^+^ or Na^+^ in its central cavity. Intervening nucleotides in such an intramolecular assembly will form loops connecting the G‐columns of the G4 core structure. Propeller loops link two adjacent columns with the same 5’‐3’ backbone orientation, whereas lateral and diagonal loops connect neighboring or distal G‐columns of antiparallel orientation, respectively.

With the formation of three loops in a canonical quadruplex with non‐interrupted G‐columns, there are 3^3^=27 theoretical loop combinations giving rise to different topologies. Additional topologies come from a clockwise (+) or anti‐clockwise (‐) progression of propeller and lateral loops when placed in a common frame of reference as previously proposed.^[5]^ For characterizing and discriminating the various folds, a simple descriptor composed of the type and progression of consecutive loops has been suggested.[^5^, ^6^] Thus, abbreviating lateral, propeller, and diagonal loops with ‘l’, ‘p’, and ‘d’, respectively, the minimalistic designation (‐pd+l) identifies a quadruplex topology with a first propeller loop running counter‐clockwise, a central diagonal loop, and a third lateral loop running clockwise. Clearly, only a fraction of the theoretical topologies can be realized for mechanical and steric reasons and fragment‐based modeling studies have identified 14 topologies that may form under appropriate conditions.[^7^, ^8^]

**Figure 1:**
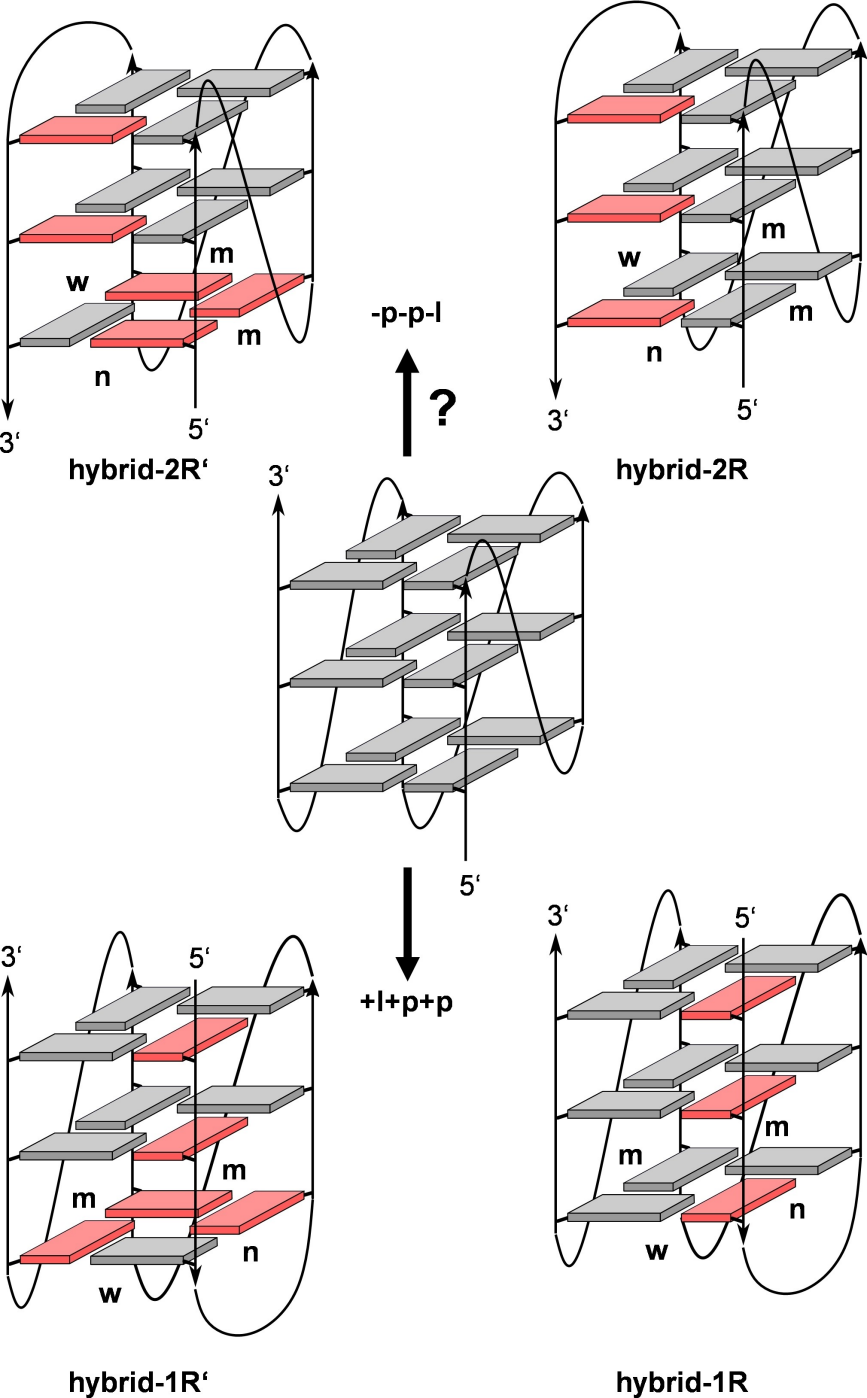
Topology of a parallel quadruplex (center) and possible (3+1) hybrid structures formed by flipping the fourth G‐tract (top) and the first G‐tract (bottom); n, m, and w denote narrow, medium, and wide groove, respectively.

G‐quadruplexes of a (3+1) hybrid‐type with one antiparallel and three parallel G‐columns are a recurrent G4 structural motif and typically represent a hybrid‐1 (‐p‐l‐l) or a hybrid‐2 (‐l‐l‐p) topology. Recently, we have shown refolding of a parallel all‐*anti* G4 into a novel (3+1) hybrid quadruplex termed hybrid‐1R with a (+l+p+p) loop progression, exploiting base complementarity of 5’‐ and 3’‐overhang sequences to form a duplex smoothly extending at one face of the rearranged G4 core.^[9]^ This topology, associated with a switch from a first propeller into a lateral loop, could be further enforced by the specific incorporation of *syn*‐favoring 8‐bromo‐guanosine (^Br^G) nucleotides, known to be powerful tools for conformational transitions.^[10]^ In the present studies, we aimed at the formation of another (3+1) hybrid species with a (‐p‐p‐l) topology starting from a parallel fold (Figure 1). Although a corresponding fold is highly ranked in a topology distribution chart of model G‐quadruplexes built from reported fragments, it has not been experimentally verified to date.[^7^, ^8^] Such a species may thus not only provide insight into critical interactions upon refolding but may also expand the known topological diversity of G‐quadruplex structures for future use in the increasing number of G4‐based applications.

### Results

The sequence design of a putative ‐p‐p‐l G‐quadruplex is based on the parallel *c‐myc* quadruplex, previously characterized in detail.^[11]^ Whereas a single nucleotide largely restricts folding of the 1‐nt intervening sequence into a propeller‐type loop, 3‐nt and 4‐nt lateral loops spanning the wide groove have most frequently been observed in fragment‐based modeling studies for a ‐p‐p‐l topology.^[7]^ We therefore started with sequence *L113* comprising a 1 : 1 : 3 nucleotide loop length arrangement. As shown in Figure 1, refolding through a switch from a propeller into a lateral loop results in a flip of the fourth G‐tract but is also associated with a relocation of the 3’‐overhang to the 5’‐face of the quadruplex core. Additionally, *anti*→*syn* conversions are enforced for some G residues to maintain G‐quartet formation and these will depend on the glycosidic bond angle pattern along the inverted strand. To probe the impact of differently modified sequences on these structural rearrangements, the fold for a total of 12 oligonucleotides was analyzed in more detail. These include variants with different loop sequences, some of which allow for a stem‐loop structure, as well as 8‐bromo‐2’‐deoxyguanosine modified analogues (Table 1).

**Table 1:** Sequences with UV melting temperatures *T*_m_.^[a,b]^

Name	Sequence	*T*_m_ [°C]
*L113*	TGA GGG T GGG T GGG TCA GGG TAA	62.0±0.2
*L112‐B12*	TGA GGG T GGG T GGG TC **BB**G TAA	59.2±0.6
*L114‐B12*	TGA GGG T GGG T GGG ACTT **BB**G TAA	50.7±0.6
*L113‐B12*	TGA GGG T GGG T GGG TCA **BB**G TAA	55.0±0.6
*L113‐B23*	TGA GGG T GGG T GGG TCA G**BB** TAA	54.3±0.7
*L113‐B13*	TGA GGG T GGG T GGG TCA **B**G**B** TAA	54.0±1.1
*L113‐B123*	TGA GGG T GGG T GGG TCA **BBB** TAA	59.7±0.1
*L11GC*	TGA GGG T GGG T GGG *GCGCGCAGCGC* GGG TAA	54.4±0.4
*L11AT*	TGA GGG T GGG T GGG *ACGCGCAGCGT* GGG TAA	59.8±0.9
*L11AT−B12*	TGA GGG T GGG T GGG *ACGCGCAGCGT* **BB**G TAA	52.4±0.3
*L11AT−B13*	TGA GGG T GGG T GGG *ACGCGCAGCGT* **B**G**B** TAA	53.5±0.7
*L11AT−B23*	TGA GGG T GGG T GGG *ACGCGCAGCGT* G**BB** TAA	53.4±0.6

[a] Loop nucleotides are underlined and complementary sequences forming putative stem‐loop structures are written in italic; **B**=8‐bromo‐2‘‐deoxyguanosine. [b] Average values with standard deviations from three independent measurements taken at 295 nm in 10 mM potassium phosphate buffer, pH 7.

#### Circular dichroism (CD)

**Figure 2:**
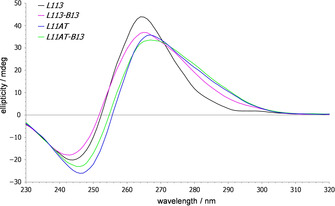
CD spectra of *L113*, *L113‐B13, L11AT*, and *L11AT‐B13*. Spectra were acquired at 20 °C on DNA samples (5 μM) in 10 mM potassium phosphate buffer, pH 7.

CD spectra of all sequences exhibit a signature typical of quadruplexes with exclusive homopolar G‐quartet stacking, i. e., a positive band at 265 nm and a negative band at 245 nm (Figure 2, for a compilation of all spectra see Figure S1). A noticeable asymmetry towards longer wavelength of the positive band and a slightly red‐shifted maximum is generally observed for all sequences bearing third loops with Watson‐Crick complementary bases. This points to the formation of a B‐type duplex extrusion and a B‐DNA signature superimposed on the G4 CD spectral features. Of note, CD spectra are compatible with a parallel as well as a (3+1) hybrid topology harboring a single all‐*syn* G‐tract, but do not allow their mutual discrimination. However, based on the CD experimental results, preferential formation of other hybrid or antiparallel quadruplexes with G‐tetrads of different polarity to result in heteropolar stacking interactions can safely be excluded for each of the G‐rich sequences.

#### NMR spectroscopy

**Figure 3:**
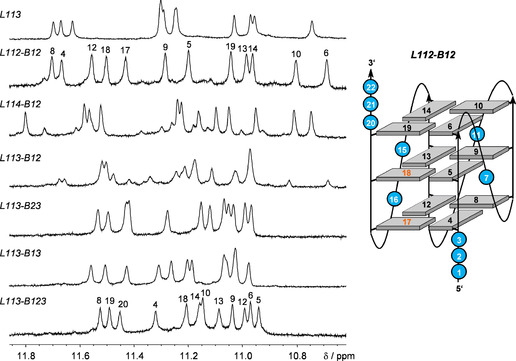
Imino proton spectral region of G4‐forming sequences without a putative duplex stem‐loop structure; residue assignments are given for *L112‐B12* and *L113‐B123*. Topology of the *L112‐B12* quadruplex with residue numbers (in orange for ^Br^G analogues) is shown on the right.

Initially, the impact on G4 folding of loop length and of *syn*‐favoring G analogues in different positions of the fourth G‐column was assessed by a comparison of imino proton NMR spectral regions (Figure 3). Due to their engagement in hydrogen bonds within a G‐tetrad, twelve guanine imino resonances in slow exchange with the solvent are expected for a three‐layered G‐quadruplex. In fact, the observation of about twelve major imino signals for each of the sequences lacking base complementarity in a longer third loop attests to their formation of a G‐quadruplex with three stacked tetrads. A closer look over all spectra reveals two different patterns of imino signals, suggesting two different topologies depending on the sequence. One pattern, covering a wider spectral range is shared by *L113* and *L112‐B12*, whereas the other pattern is shared by *L113‐B23*, *L113‐B13*, and *L113‐B123*. The presence of a major and minor set of imino resonances for *L114‐B12* and *L113‐B12* suggests coexistence of both topologies, albeit in different molar ratios.

Sequences *L112‐B12* and *L113‐B123*, each representing one type of fold, were chosen for an initial determination of topologies. Also, their well‐resolved imino proton resonances favor a more detailed NMR structural analysis. For *L112‐B12*, a straightforward identification of a standard parallel all‐*anti* topology was based on the absence of *syn*‐guanosines but also on conspicuous NOE contacts between H8 of 3’‐terminal A22 and G6 as well as G19 imino protons located at the G‐core face opposite to the 5’‐overhang (Figure S2). Thus, all guanine H8‐C8 cross‐peaks of *L112‐B12* are located in a ^1^H‐^13^C HSQC spectral region typical for an *anti* glycosidic conformation (Figure S3A) and this is further supported by the lack of strong intranucleotide H8‐H1’ and associated weak H8‐H2’/H2” contacts in a NOESY spectrum, anticipated for *syn*‐residues (for assignments and a compilation of *L112‐B12* chemical shifts see Table S1).

**Figure 4:**
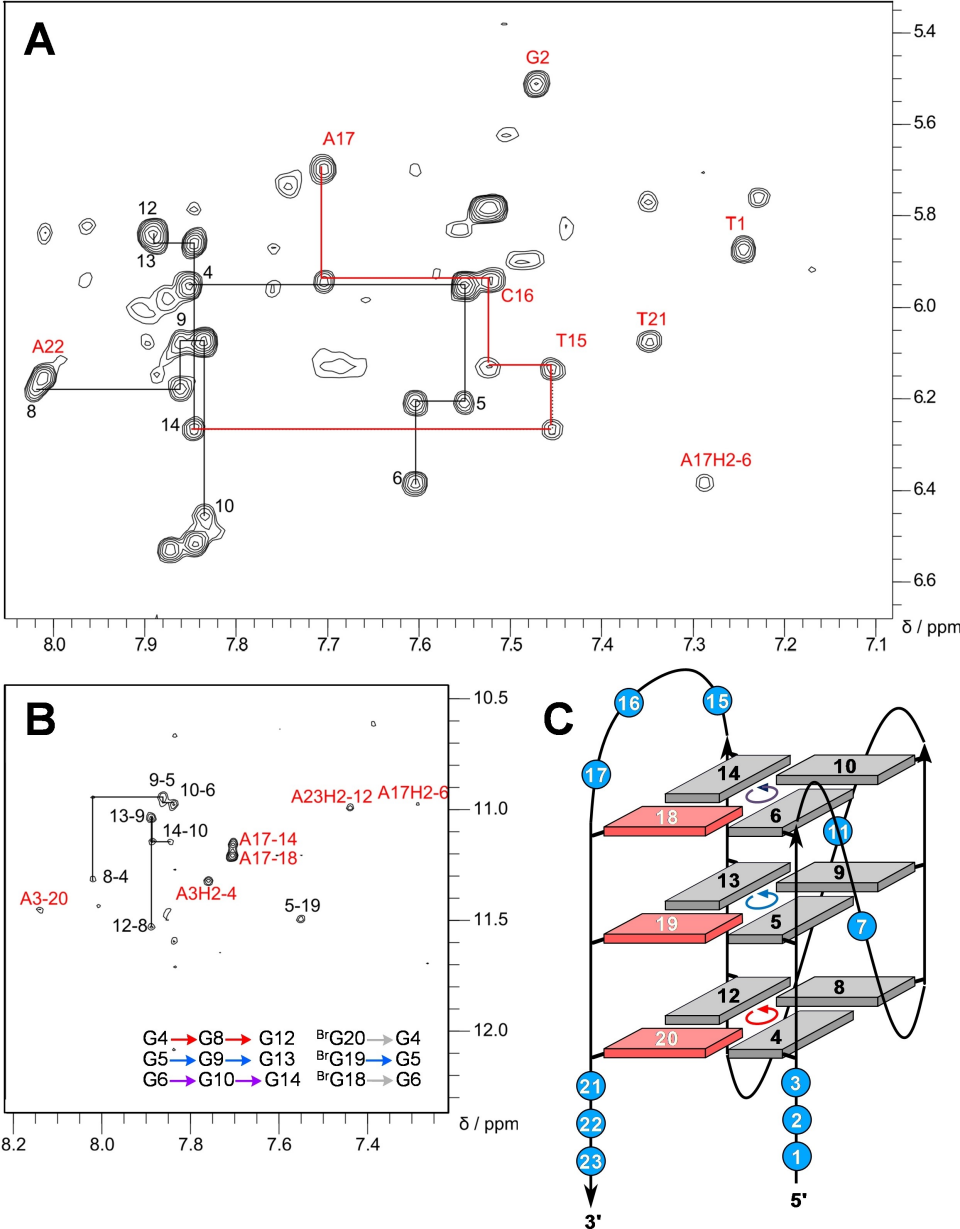
2D NOE spectral regions of *L113‐B123*. (A) H8/6(ω_2_)‐H1’(ω_1_) cross‐peaks with sequential contacts traced along the G‐core (black) as well as along the 3‐nt third loop (red). (B) Intra‐ and inter‐tetrad H8(ω_2_)‐H1(ω_1_) cross‐peaks and additional contacts involving overhang residues and A17 of the 3‐nt loop labeled in red. Tetrad polarities as determined from intra‐tetrad NOE contacts are summarized at the bottom (grey arrows indicate missing contact). (C) Topology of the *L113‐B123* quadruplex with residue numbers and tetrad polarities indicated; *syn*‐^Br^G analogues are shown in red with their residue numbers in white.

The identification of *syn* residues for a topological evaluation of *L113‐B123* is restricted due to the absence of G H8 protons in the fully ^Br^G‐modified fourth G‐tract. However, various NOE contacts of residues in the third 3‐nt loop strongly suggest its lateral progression with a flipped antiparallel fourth G‐column in a hybrid‐type fold. These include sequential contacts along the loop and cross‐peaks between H2/H8 of loop residue A17 with three out of four imino protons in the outer tetrad opposite the 5’‐terminus (Figure 4; for assignments and a compilation of *L113‐B123* chemical shifts see Table S2).

A close structural similarity of *L113* with *L112‐B12* as well as of *L113‐B13* and *L113‐B23* with *L113‐B123*, initially suggested through corresponding signal patterns in the imino proton spectral region, was confirmed by a more detailed analysis of corresponding NOESY and ^1^H‐^13^C HSQC spectra (not shown). Thus, a single *syn*‐G compatible with a ^Br^G‐disubstituted all‐*syn* column could be identified in *L113‐B13* and *L113‐B23*. Also, NOE contacts from loop and 3’‐overhang residues to outer tetrad resonances in all sequences could either be traced to a third propeller loop and overhang sequences on opposite sides of the G‐core (*L113*) or to a third lateral loop with both termini facing each other (*L113‐B13* and *L113‐B23*).

Assignments for *L113‐B12* and *L114‐B12*, exhibiting two coexisting species based on their imino proton spectral region, again relied on spectral comparisons with both the *L112‐B12* parallel quadruplex and the *L113‐B123* hybrid‐type G4. Thus, a superposition of HSQC and NOESY spectral regions of *L113‐B12* with those of *L112‐B12* and *L113‐B123* clearly demonstrated its folding into a major hybrid‐type topology with the observation of a single *syn*‐G and a minor parallel topology with a population ratio estimated to be about 7 : 3 (Figure S3). In contrast, the major species of *L114‐B12* can likewise be assigned to a parallel fold with cross‐peak patterns very similar to *L112‐B12* (Figure S4). A decrease in the melting temperature for *L114‐B12* by almost 10 °C when compared to *L112‐B12* can thus directly be attributed to a destabilizing effect from its longer 4‐nt propeller loop (Table 1). With a population of only about 20 %, minor peaks of a putative (3+1) hybrid fold in *L114‐B12* spectra do not allow their unambiguous assignments.

#### Quadruplexes with a duplex stem‐loop

To further promote a switch from the third propeller into a lateral loop with formation of a hybrid‐type quadruplex, the loop sequence was expanded to enable hairpin formation through Watson‐Crick base pairing. This should allow for a coaxial orientation with contiguous base stacking interactions of a putative hairpin‐type lateral loop and the quadruplex helix.^[12]^ Initially, two sequences *L11AT* and *L11GC* with a 11‐nt third loop capable of stem‐loop formation and with AT or GC complementary bases adjacent to the G‐core were tested for their favored topology.

**Figure 5:**
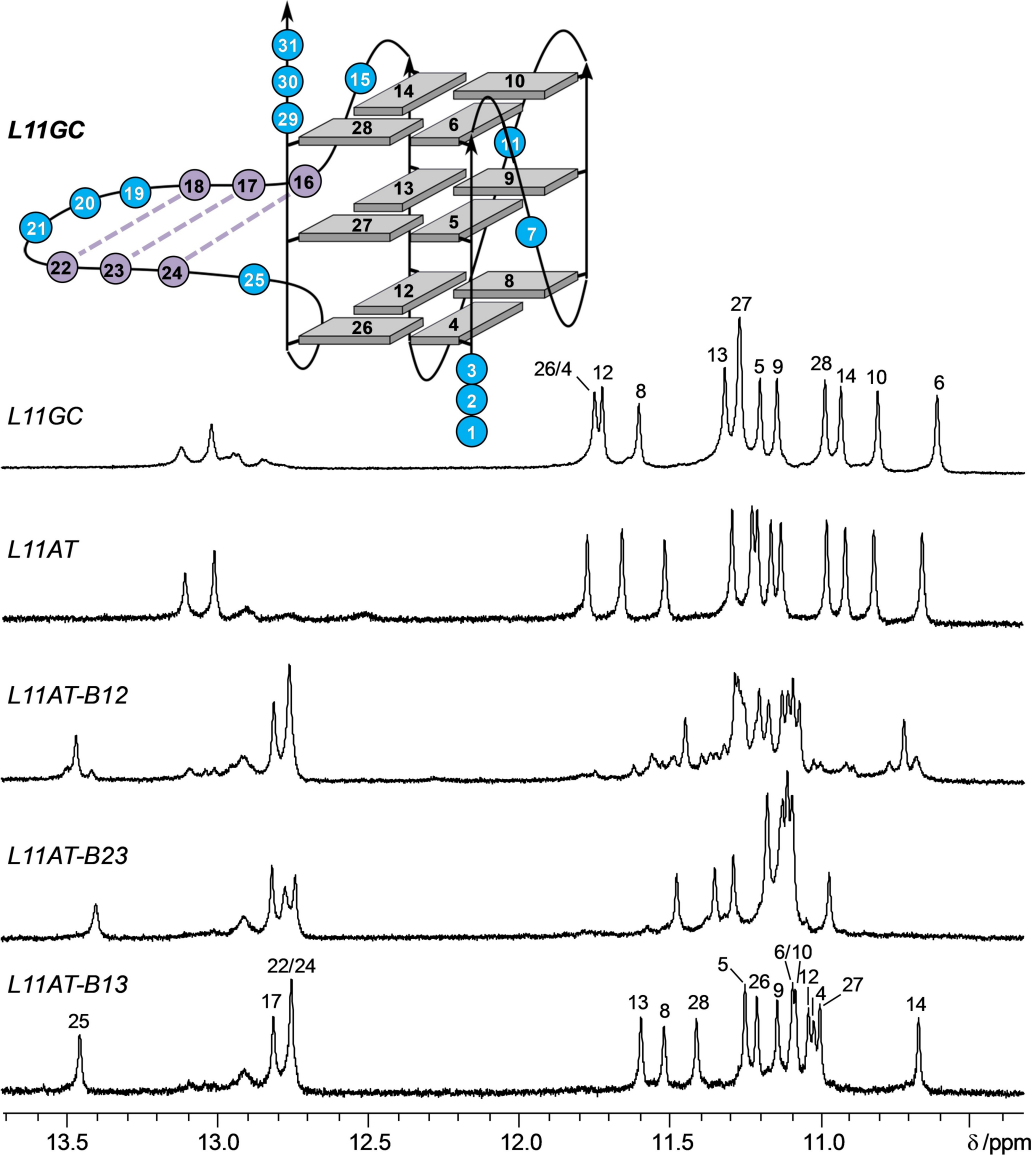
Imino proton spectral region of G4‐forming sequences with a duplex stem‐loop structure; residue assignments are shown for *L11GC* and *L11AT‐B13*. Topology of the *L11GC* quadruplex with residue numbers is shown on top; GC Watson‐Crick base pairing within the 11‐nt propeller loop is indicated by broken lines.

Inspection of their guanine Hoogsteen imino proton spectral region between 11.8 and 10.6 ppm reveals a closely similar signal distribution to *L113*, indicating a parallel G4 topology for both sequences (Figure 5). Additional downfield‐shifted resonances between 12.8 and 13.2 ppm can be attributed to imino protons engaged in GC Watson‐Crick base pairs exchanging with the solvent at different rates. Notably, there is no indication of a stable AT Watson‐Crick base pair at the quadruplex‐duplex junction in *L11AT* because spectra lack an expected imino signal at low field. These observations are consistent with base pairing in a propeller loop, enforcing an orthogonal orientation of duplex and quadruplex helices with unpaired bases at the junction.^[12]^ Featuring additional base pairs within the loop devoid of additional stacking interactions with outer G‐tetrads, G4 stability decreases with the longer 11‐nt loop compared to *L113* with its 3‐nt loop (Table 1). A more detailed analysis of *L11GC* spectra confirms its parallel fold characterized by (i) the absence of *syn*‐Gs in HSQC spectra (not shown), (ii) NOE contacts between residues of the 5’‐ and 3’‐overhang with opposite outer tetrads, and (iii) the absence of sequential contacts between loop residues and the G‐core (Figure S5, for assignments and a compilation of *L11GC* chemical shifts see Table S3).

**Figure 6:**
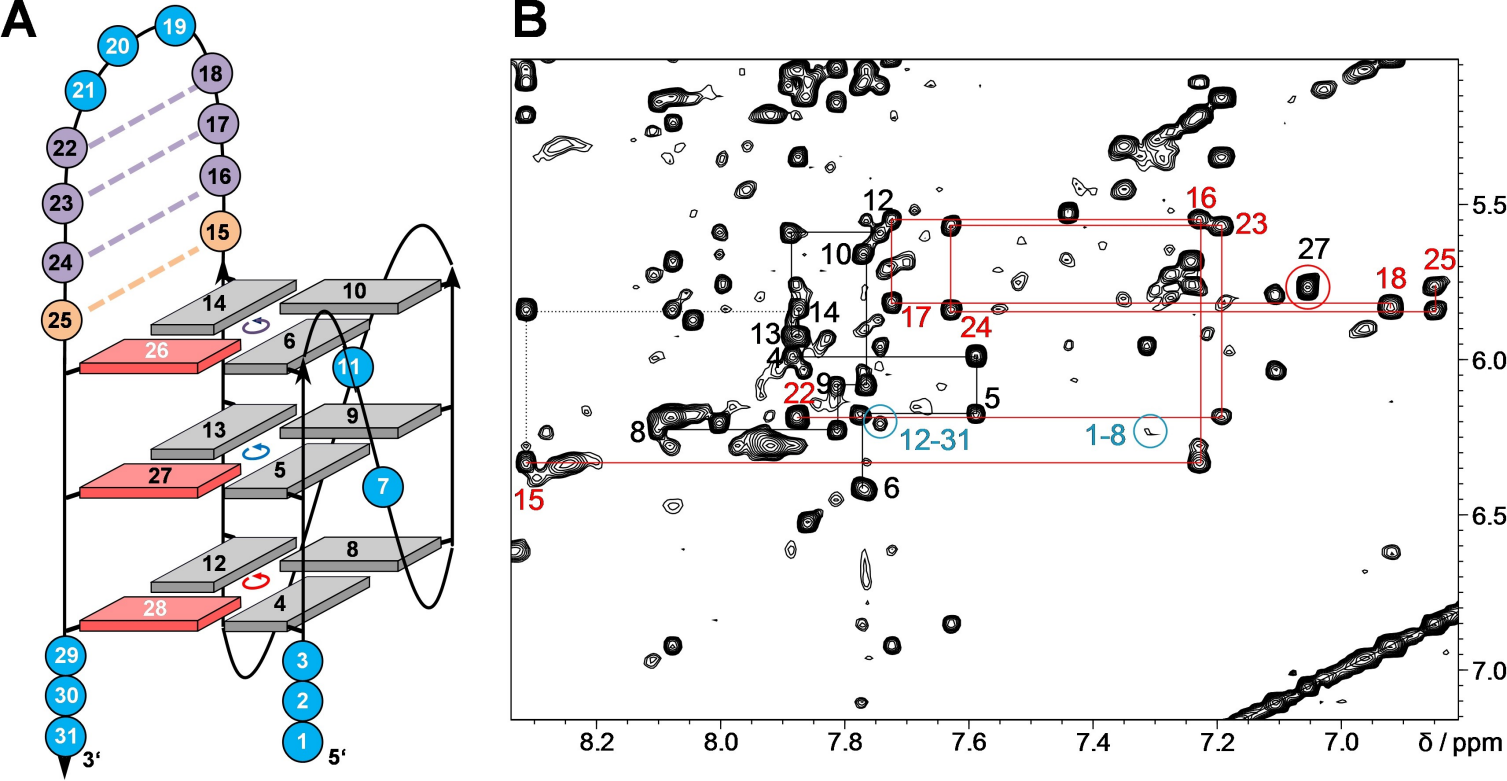
(A) Topology of the *L11AT‐B13* quadruplex with residue numbers and tetrad polarities indicated; AT and GC Watson‐Crick base pairs within the 11‐nt stem‐loop are indicated by broken orange and violet lines, respectively; *syn*‐^Br^G analogues are shown in red with white residue numbers. (B) 2D NOESY spectrum of *L11AT‐B13*. H8/6(ω_2_)‐H1’(ω_1_) connectivities are traced along the G‐columns (black) and the duplex stem (red) with an intra‐residual contact of *syn*‐G27 circled in red; note a sequential NOE contact between residue 14 and 15 at the quadruplex‐duplex interface and cross‐peaks (circled in blue) between residues of both 5’‐ and 3’‐overhangs with the 5’‐outer tetrad.

Adopting the same parallel fold as *L11GC*, we selected the *L11AT* quadruplex for additional ^Br^G substitutions with a putative AT base pair at the quadruplex‐duplex junction serving as a convenient probe for refolding into a hybrid‐type topology. In fact, the pattern of Hoogsteen imino protons together with the observation of an additional downfield‐shifted AT Watson‐Crick imino resonance at ∼13.5 ppm suggests a non‐parallel fold for *L11AT‐B12*, *L11AT‐B13*, and *L11AT‐B23* harboring two ^Br^G modifications in their 3’‐terminal G‐tract. Formation of a lateral hairpin structure with an AT base pair at the quadruplex‐duplex junction is corroborated by sequential base‐sugar contacts which can be traced along the duplex domain into the adjacent G‐tracts (Figure 6). Also, folding into a hybrid‐type structure for all three dual‐modified sequences is suggested by the observation of a single *syn*‐G in HSQC and NOESY spectra (see below). Interestingly, *L11AT‐B13* and *L11AT‐B23* form a single G4 species with about the same melting temperature as those of *L113‐B13 and L113‐B23* with short 3‐nt lateral loops, excluding significant synergistic effects of duplex and quadruplex domains (Table 1). For *L11AT‐B12*, another structure coexists with the major hybrid structure. However, with a population of only about 10 %, this minor species escapes a more detailed structural investigation. In the following, we focused on *L11AT‐B13* for a three‐dimensional structure determination due to its superior signal dispersion with minimal signal overlap.

#### Structure determination of L11AT‐B13

Ample evidence from initial NMR spectral analyses points to a (3+1) hybrid fold for *L11AT‐B13* (Figure 6A). Based on such a topology, complete resonance assignments followed standard procedures. Continuous H8/H6‐H1’/H3’ and H8/H6‐H2’/H2” NOE walks can be traced along all G‐tracts except for the dual‐modified G‐column at the 3’‐end. Likewise, corresponding sequential NOE cross‐peaks connect residues within the duplex stem‐loop as well as A15 and G14 at the duplex‐quadruplex interface (Figure 6B). In addition, residues of the duplex stem comprising four Watson‐Crick base pairs exhibit well‐resolved sequential H8/6‐H8/6 cross‐peaks (Figure S6A). Non‐modified G27 within the 3’‐terminal G‐tract is identified through its *syn* conformation, exhibiting strong intra‐nucleotide H8‐H1’ and weak H8‐H2’/H2” cross‐peaks in NOESY spectra acquired with short mixing times (Figure S7A). A single unmodified *syn*‐G27 is further corroborated by its characteristic C8‐H8 cross‐peak chemical shift in a ^1^H‐^13^C HSQC spectrum (Figure S7B).

**Figure 7:**
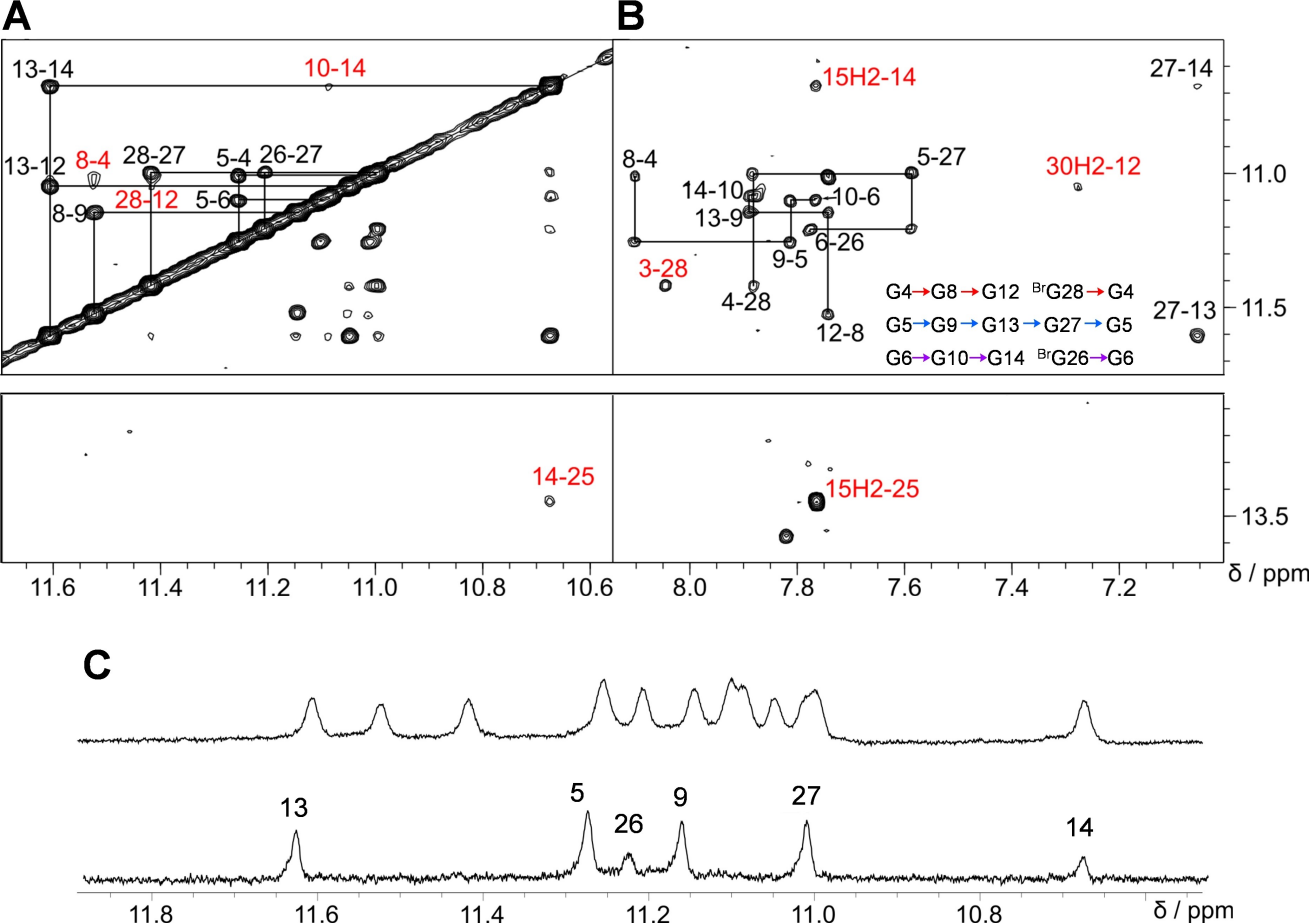
(A) Imino‐imino 2D NOE spectral region of *L11AT‐B13* with sequential cross‐peaks and additional contacts at the junction or within tetrads labeled in red. (B) Intra‐ and inter‐tetrad H8(ω_2_)‐H1(ω_1_) cross‐peaks; contacts at the quadruplex‐duplex junction, between 5’‐ and 3’‐overhang residues with the 5’‐outer tetrad, and within the AT Watson‐Crick base pair are labeled in red. Tetrad polarities as determined from intra‐tetrad NOE contacts are summarized as inset. (C) H_2_O‐D_2_O exchange experiments. Imino proton spectral region of *L11AT‐B13* acquired in H_2_O‐D_2_O (9 : 1) (top) and shortly after drying and redissolving in 100 % D_2_O (bottom); imino protons for residues of the central tetrad and, albeit to a smaller extent, of G14 and ^Br^G26 at the quadruplex‐duplex junction are protected from fast solvent exchange.

Sequential imino‐imino NOE contacts link G residues along the same G‐column (Figure 7A). Notably, whereas inter‐tetrad cross‐peaks of imino protons to nearest H2’/H2” protons located in residues on the 3’‐adjacent G‐tract are characteristically weak and often unobservable for parallel all‐*anti* G‐columns, conspicuous sequential H1(n)‐H2’/H2”(n‐1) cross‐peaks, typical for *syn*‐residues,[^9^, ^13^] were observed along the fourth G‐tract and comprise H2’/H2” of 5’‐adjacent T25 of the lateral stem‐loop (Figure S6B). Finally, H1‐H8 NOE contacts between adjacent Gs within tetrads, only interrupted by missing H8 protons of the two ^Br^G analogues in the outer quartets, identify the direction of Hoogsteen hydrogen bonds and demonstrate the same polarity for all tetrads (Figure 7B).

There are several NOE contacts at the quadruplex‐duplex interface. These include NOE cross‐peaks of G14 with A15, T25 with ^Br^G26, but also of G6 and G14 with T25, suggesting a well‐defined quadruplex‐duplex junction (see Figures 7B and S6). This is also corroborated by H_2_O‐D_2_O exchange experiments, showing protection from solvent exchange for G residues of the central tetrad but also slower exchange for G14 and G26 at the quadruplex‐duplex junction consistent with continuous stacking (Figure 7C). Few contacts between overhang residues and the adjacent G‐tetrad such as G12 H1‐A30 H2, G28 H1‐A3 H8, G12 H8‐A31 H1’, and a weak cross‐peak between G8 H1’ and T1 H6 point to, albeit more flexible, structured 5’‐ and 3’‐flanking sequences (Figure 6B and 7B).

H2’ and H2” resonances were stereospecifically assigned through cross‐peak intensities of intra‐nucleotide H8‐H2’/H2” and H1’‐H2’/H2” contacts in NOESY spectra acquired at short mixing times. Evaluation of vicinal H1’‐H2’ and H1’‐H2” scalar couplings in DQF‐COSY spectra demonstrated *south* sugar conformations for all residues of the quadruplex and the hairpin domain except for G‐core nucleotides G6, G13, G4, and G26 as well as for stem‐loop nucleotides C16, G24, and T25. For the latter, sugar conformations remained ambiguous due to heavy signal overlap (Figure S8).

**Figure 8:**
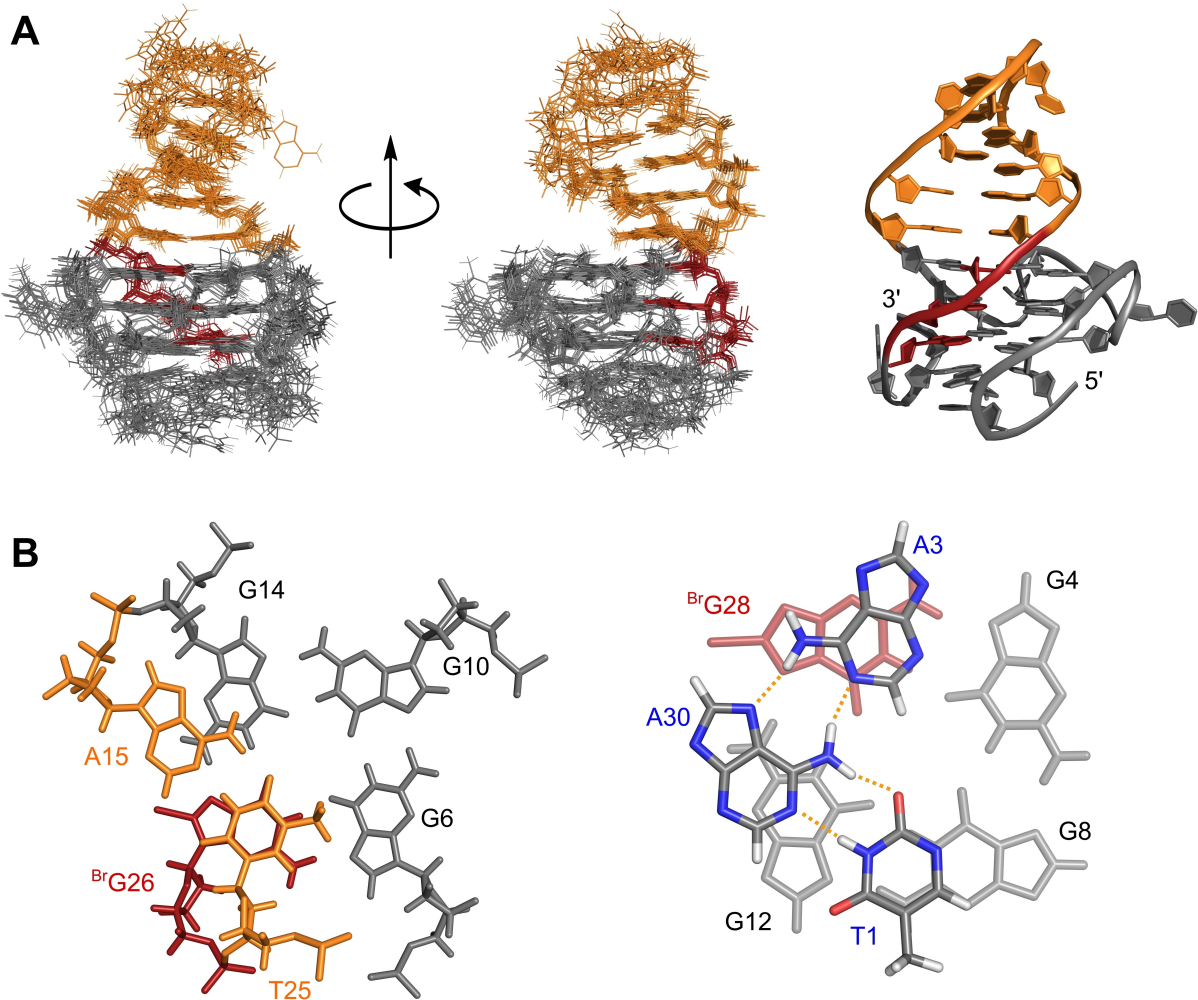
(A) Superposition of 10 lowest‐energy structures (left) and representative structure of *L11AT‐B13* (right); duplex stem‐loop is colored orange, *syn*‐residues of the G‐core are colored red. (B) Q−D junction (left) and possible capping structure stacked onto the 5’‐tetrad (right); duplex and G‐core residues are colored in orange and red/grey, respectively; 5’‐ and 3’‐ overhang residues as part of a planar capping structure observed in the lowest energy structure are colored by element with potential hydrogen bonds traced by dotted lines.

For subsequent structure calculations, NOE‐derived distance restraints but no sugar torsion angle restraints were employed (for structural statistics see Table S5). The quadruplex adopts a novel (‐p‐p‐l) topology with two propeller followed by a lateral loop running in a counter‐clockwise direction (Figure 8A). We term this quadruplex fold hybrid‐2R in analogy to the previously reported hybrid‐1R quadruplex with a (+l+p+p) topology derived from hybrid‐1 structures by formally substituting propeller for lateral loops and vice versa. Looking at a superposition of the ten lowest‐energy structures, the G‐core, the 4‐bp duplex stem, and especially the quadruplex‐duplex interface are well‐defined (Figure 8A). In contrast, propeller loops and the loop of the duplex hairpin are less ordered, but also 5’‐ and 3’‐overhang residues seem to experience significant flexibility. With all G residues of the three parallel G‐tracts adopting an *anti* glycosidic conformation, the antiparallel fourth G‐tract with its two ^*Br*^G modifications exclusively features *syn*‐residues.

The stem‐loop structure stacks with its terminal AT base pair coaxially on G14 and ^Br^G26 of the upper quadruplex tetrad (Figure 8B), rationalizing protection of these outer G‐core residues from fast solvent exchange as found experimentally (Figure 7C). Because the wide groove of the G‐core bridged by the duplex stem‐loop and the minor groove of a B‐type duplex are of similar width, the quadruplex‐duplex junction allows for a smooth transition without major distortions of the sugar‐phosphate backbone.

Although less defined and rather flexible, overhang sequences seem to form loose capping structures stacked below the G‐tetrad. Thus, corroborated by 2D NOE data, all calculated low‐energy structures show stacking of A3 on either G4 or ^Br^G28 and stacking of T1 on G8 is observed in 7 out of 10 structures. Also, various alignments of flanking bases are observed in the final structures with the lowest energy structure featuring an A⋅A⋅T base triad involving 5’‐flanking residues T1 and A3 as well as 3’‐flanking residue A30 (Figure 8B). Hydrogen bonds in such a base triad lack final confirmation by experimental data, pointing to a high flexibility with fast dissociation rates in rather short‐lived base arrangements.

#### Probing refolding through ligand binding

Refolding of the parallel *L11AT* quadruplex into a hybrid‐type quadruplex upon ^Br^G substitutions in its fourth G‐tract results in a coaxial stacking of the stem‐loop duplex onto the outer G‐tetrad with the formation of a quadruplex‐duplex interface. Quadruplex‐duplex (Q−D) junctions may serve as specific recognition elements for ligands.[^14^, ^15^] In fact, a corresponding Q−D junction has recently been shown to constitute a favorable binding site for a ligand termed PIQ which is based on a phenyl‐substituted indoloquinoline heterocyclic ring system.^[16]^ Isothermal titration calorimetry (ITC) was initially employed to probe PIQ binding to the novel hybrid‐2R G*‐*quadruplex with its stacked hairpin lateral loop. To eliminate any putative impact of a bromo substituent at the quadruplex‐duplex interface, the *L11AT‐B23* analogue was used as receptor for the ligand. Also, a 20 mM potassium phosphate buffer supplemented with 100 mM KCl was used for a better compatibility with previous ITC experiments after verification by NMR spectroscopy that the topology is conserved under the higher salt concentrations (not shown). As an additional benefit of a higher ionic strength, non‐specific electrostatic interactions between the cationic ligand and the polyanionic quadruplex may efficiently be suppressed.^[17]^

**Figure 9:**
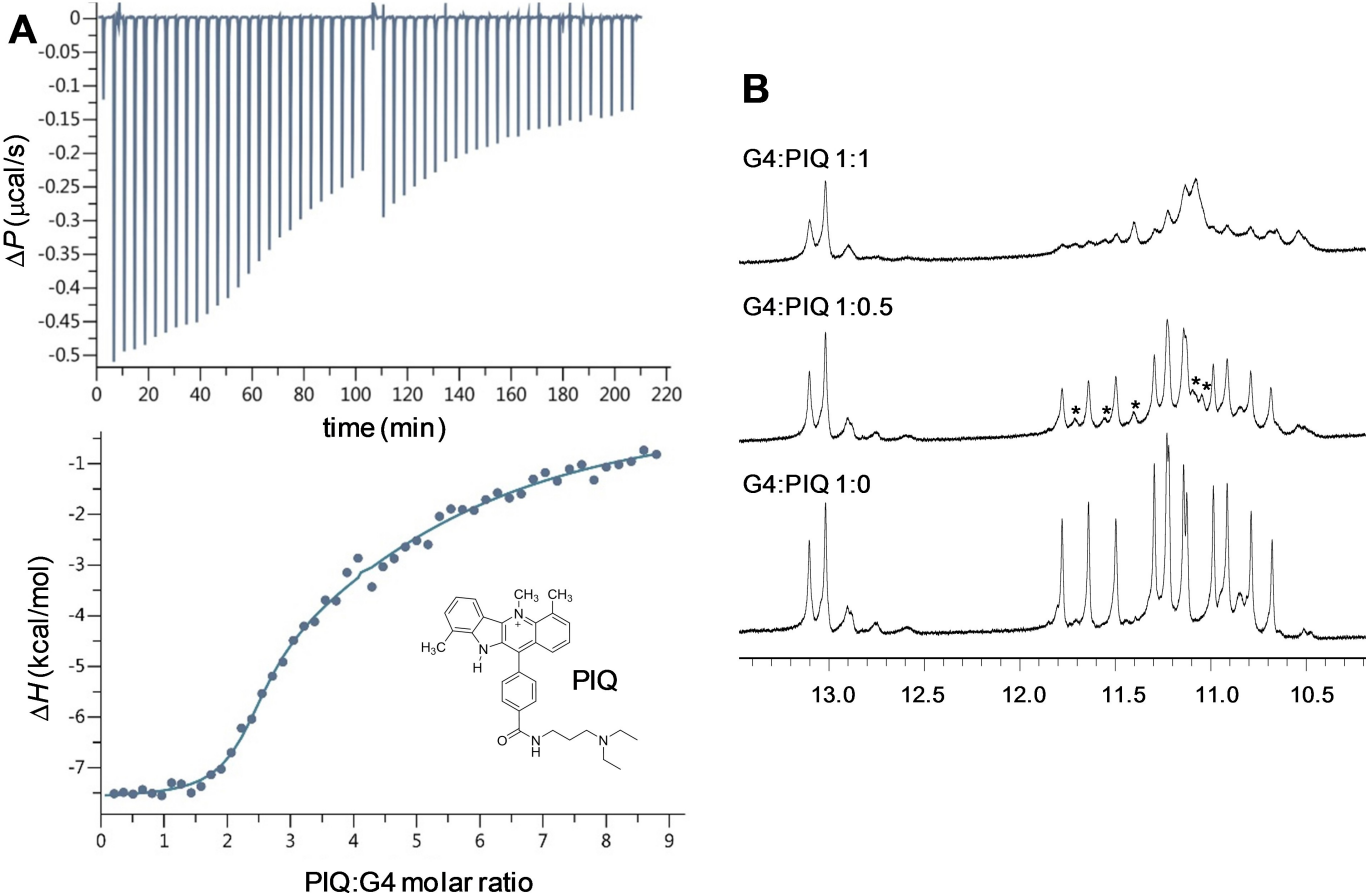
(A) Representative ITC experiment with the PIQ ligand titrated to the *L11AT‐B23* quadruplex at 40 °C in 100 mM KCl, 20 mM potassium phosphate buffer, pH 7; the upper panel shows the heat burst for every injection step with a central discontinuity due to reloading the injection syringe for covering a wider concentration range; the lower panel shows the dilution‐corrected heat versus the molar ratio with a least square fit. (B) Imino proton spectral region of the *L11AT* quadruplex titrated with the PIQ ligand at 30 °C in 10 mM potassium phosphate buffer, pH 7; some newly observed resonances after addition of 0.5 equivalent of ligand are indicated by an asterisk.

A representative ITC isotherm obtained after integration of the power output for each injection and corrections for the heats of dilution is shown in Figure 9A (bottom) for the PIQ titration to the *L11AT‐B23* receptor at 40 °C. Strong exothermic binding during initial titration steps followed by only a gradual return to baseline with ligand added in large excess points to at least two non‐equivalent binding sites of very different affinity. Fitting the data of three independent experiments with a two‐site model yielded an association constant *K*_a_ for high‐affinity binding of (4.4±3.1)×10^6^ M^−1^ and a binding molar enthalpy Δ*H*° of −8.4±0.7 kcal mol^−1^. In contrast, second binding with *K*_a_ of ∼3×10^4^ M^−1^ is lower by two orders of magnitude and likely suggests PIQ binding to the duplex stem‐loop.

Recently, an association constant *K*_a_ of 2×10^6^ M^−1^ and a binding enthalpy Δ*H*° of −6.3 kcal mol^−1^ was determined under identical conditions for PIQ binding to a *c‐myc*‐derived quadruplex with the same flanking residues as in *L11AT‐B23*.^[17]^ Lacking a duplex stem‐loop, the latter G4 is forced into a parallel topology through its short 1‐ and 2‐nt loops. Notably, extending one overhang sequence of the *c‐myc* G4 to form a regular Watson‐Crick duplex stacked on an outer tetrad yielded a stronger and also a more exothermic association, attributable to a favored PIQ binding at the newly formed Q−D junction.^[16]^ Based on these results and on the noticeable increase in affinity and exothermicity when going from the non‐extended *c‐myc* to the hybrid‐type *L11AT‐B23* quadruplex, high‐affinity binding to the latter is proposed to occur at the Q−D junction of *L11AT‐B23* as being the most favorable binding site.

Because such a binding preference should promote refolding from a parallel into the hybrid topology in the presence of the ligand, additional NMR titrations of the parallel non‐modified *L11AT* quadruplex with PIQ were performed (Figure 9B). With addition of ligand in sub‐stoichiometric amounts, a second set of imino resonances emerges. Unfortunately, all G4 imino signals experience significant line‐broadening due to dynamic exchange processes upon continued ligand titration. As a result, the newly formed complex resonances could not be analyzed and assigned to a particular topology. On the other hand, a single most downfield shifted AT Watson‐Crick imino resonance is expected at the Q−D junction for a hybrid‐2R topology. Disregarding severe broadening effects, the absence of a corresponding AT imino seems to argue against a ligand‐induced refolding. As a consequence, the gain in binding free energy for the PIQ ligand seems to be insufficient to close the energy gap between the interconverting topologies.

## Discussion

Previous studies have succeeded in switching the first propeller loop of a parallel quadruplex to a lateral loop with the formation of a (+l+p+p) topology termed hybrid‐1R. To access a novel (‐p‐p‐l) hybrid‐type G4 topology, the present work aimed at a corresponding rearrangement of the third loop to invert the last G‐tract of a parallel G4. Although a flip of either the 5’‐ or 3’‐terminal G‐tract seems largely similar, it imposes different steric constraints. Whereas a 5’‐3’ backbone inversion of the first G‐column in a parallel quadruplex will result in a lateral loop bridging a narrow groove and two overhang sequences facing each other in a wide groove, inversion of the fourth G‐column will be accompanied by a lateral loop spanning a wide groove and 5’‐ and 3’‐termini facing each other in a narrow groove. As a result, a 2‐nt intervening sequence between the third and fourth G‐tract was found to be too short to allow formation of a lateral loop for the present sequences, although 2‐nt lateral loops bridging a wide groove have been reported.[^18^, ^19^] Here, a 3‐nt loop was shown to be favored for the folding into a (‐p‐p‐l) topology whereas an even longer and less structured 4‐nt loop proved to be less efficient.

Because the width of a B‐DNA minor groove closely matches the width of a quadruplex wide groove, a lateral Watson‐Crick stem‐loop is expected to smoothly extend coaxially from the quadruplex core with continuous base stacking.^[12]^ In contrast, short distances will prevent overhang bases next to the G‐core from forming a regular Watson‐Crick base pair. In line with these considerations, a more efficient strand flip was attempted by replacing a short lateral loop with a putative stem‐loop forming sequence in the present studies. In fact, a mild promotion of refolding into a lateral loop through coaxial stem‐loop formation could be observed through a moderate increase in the population of a rearranged hybrid structure in case of *L11AT‐B12* when compared to *L113‐B12*. However, whereas base pairing between the 5’‐ and 3’‐overhang were previously found to induce a partial flip of the first G‐column even without inserting *syn*‐favoring G analogues to form a hybrid‐1R topology,^[9]^ base pairing within a lateral loop as in *L11AT* and *L11GC* failed to enforce refolding into a corresponding hybrid‐2R fold without support from additional ^Br^G modifications. The rather small impact of a stem‐loop on refolding may be attributed to only partial disruptions of base pairs in a parallel G4. Thus, a propeller loop connecting two faces of the G‐core can also accommodate a B‐type hairpin, yet an orthogonal orientation between quadruplex core and a duplex stem‐loop in such a case excludes base stacking at the interface and also prevents bases proximal to the junction from forming a Watson‐Crick base pair.

Having enforced a complete refolding by the incorporation of two ^Br^G modifications into the fourth G‐tract, formed hybrid structures with a 4‐bp lateral stem‐loop exhibit unaltered thermal stabilities when compared to corresponding hybrid quadruplexes with only a 3‐nt lateral loop (Table 1). This is in contrast to previous observations on a two‐tetrad antiparallel quadruplex, showing a moderate increase in melting temperature upon additional stacking of a lateral duplex hairpin onto an outer G‐tetrad.^[20]^ Apparently, there is no significant stability gain for the hybrid‐type quadruplex from stacking interactions of an additional base pair with its outer tetrad. On the other hand, the 11‐nt hairpin‐type propeller loop linked by two unpaired bases to opposite faces of the G‐core in *L11AT* and *L11GC* seems to compromise G4 stability. This is suggested by the lowering of melting temperatures when going from *L113* to *L11AT* and *L11GC*, with all three non‐modified sequences exclusively folding into a parallel topology. Thus, a favored G‐tract inversion in the presence of a base‐paired loop as implied by a comparison of *L113‐B12* and *L11AT‐B12* may rely on a destabilization through the long propeller loop in a parallel G4 rather than on additional G4 stabilization through the stacking of a lateral stem‐loop. Clearly, the latter implies that relative melting temperatures also reflect relative thermodynamic stabilities at ambient temperatures. Alternatively, formation of different topologies may also be kinetically controlled and a remarkable increase in folding kinetics has recently been reported for a parallel topology in the presence of a fast forming duplex stem‐loop, thought to bring G‐tracts closer to each other.^[21]^ Whereas in the latter studies the double‐helical domain was linked by non‐complementary unpaired bases to the G‐core, initial formation of a hairpin structure with a terminal base pair directly adjoining G‐tracts may conceivably promote fast folding into a hybrid structure through its duplex stem‐loop mediated preorganization.

Structural analysis of the formed (‐p‐p‐l) hybrid‐2R quadruplex demonstrates a fourth G‐column exclusively comprising all‐*syn* residues to yield four G(*anti*)⋅G(*anti*)⋅G(*anti*)⋅G(*syn*) tetrads of the same polarity. Given more favorable stacking interactions in *syn*‐*anti* and *anti*‐*anti* steps when compared to *syn*‐*syn* and *anti*‐*syn* steps along a G‐column,[^22^, ^23^] a hybrid‐2R’ conformation with a G(*syn*)‐G(*syn*)‐G(*anti*) glycosidic bond pattern of the fourth G‐tract associated with a single G(*anti*)⋅G(*syn*)⋅G(*syn*)⋅G(*syn*) tetrad and homopolar as well as heteropolar tetrad stackings would be easily conceivable (Figure 1). This should specifically apply to *L11AT‐B12* that comprises two 5’‐positioned *syn*‐affine ^Br^G analogues and a natural G residue at the G‐tract 3’‐position. Although such a hybrid‐2R’ conformation for a sequence with substitutions at the first two positions in the last G‐tract is potentially disfavored by a larger number of non‐brominated *syn*‐G residues (3 versus 1, see Figure 1), it would harbor only a single unfavorable *syn*‐*syn* step. In fact, the presence of a *L11AT‐B12* minor species points to its folding into a coexisting parallel topology, but refolding into an additional hybrid‐type conformer with a fourth G(*syn*)‐G(*syn*)‐G(*anti*) column cannot be excluded. Thus, observation of additional low‐intensity AT Watson‐Crick imino resonances of *L11AT‐B12* are compatible with formation of a hybrid‐2R’ conformer (see Figure 5). Unfortunately, the low population prevents its unambiguous characterization through CD and NMR spectral analysis. Of note, previously reported hybrid‐1 and hybrid‐2 topologies, each forming one propeller and two lateral loops, fold into quadruplexes with a glycosidic bond angle pattern featuring one antiparallel G(*syn*)‐G(*syn*)‐G(*anti*) and three G(*syn*)‐G(*anti*)‐G(*anti*) columns.[^24^, ^25^, ^26^] Also, in flipping the first G‐column of a non‐modified parallel quadruplex through complementary overhang sequences to form a (+l+p+p) hybrid‐1R topology, quadruplexes with a first all‐*syn* tract were also shown to coexist with a corresponding hybrid‐1R’ conformation exhibiting a first G(*syn*)‐G(*syn*)‐G(*anti*) column, albeit with a lower population.

The newly characterized hybrid‐2R fold expands the topological toolbox for various G4 technological applications. On the other hand, the present studies suggest that, although sterically feasible, a (‐p‐p‐l) quadruplex topology seems disfavored in sequences lacking nucleotide analogues with specific conformational preferences. However, with an intervening sequence able to form a duplex hairpin when linking the third and fourth G‐tract, ligands selectively binding with high affinity to a quadruplex‐duplex junction as formed in a hybrid‐2R topology may trigger a corresponding rearrangement by shifting equilibria. A ligand‐induced refolding of *L11AT* from a parallel into a hybrid‐2R topology upon addition of the indoloquinoline PIQ, previously found to favor binding at a quadruplex‐duplex junction, remains questionable but highly negative binding free energies of even more specific Q−D binders may overcome energy barriers to effectively drive G4 refolding. As a consequence, hybrid‐2R topologies may also occur under *in vivo* conditions in the presence of binding metabolites or proteins without additional modifications.

## Experimental Section

### Materials and sample preparation

All oligonucleotides were synthesized by TIB MOLBIOL (Berlin, Germany) and additionally purified by precipitation with potassium acetate and ethanol. Concentration of DNA was determined in triplicate by the UV absorbance of its unfolded species at 80 °C, employing molar extinction coefficients as provided by the supplier. For optical and NMR measurements, samples were dissolved in 10 mM potassium phosphate buffer, pH 7. Concentrations of final samples were 5 μM for UV and CD measurements and ranged from 0.2 to 1 mM for NMR studies. Prior to measurements, samples were annealed by heating to 90 °C for 5 min followed by their slow cooling to room temperature. PIQ was prepared as described previously and its concentration determined spectrophotometrically by using a molar extinction coefficient ϵ_376_=22227 L ⋅ mol^−1^ ⋅ cm^−1^.^[27]^

### UV melting

Melting temperatures for the quadruplexes were determined with a Jasco V‐650 spectrophotometer equipped with a Peltier thermostat. Temperature dependent absorbances were recorded with a heating and cooling rate of 0.2 °C per minute at 295 nm. There was no hysteresis, indicating thermodynamic equilibrium conditions. Melting temperatures were determined by the minimum of a first derivative plot. In case of a poor signal‐to‐noise ratio, the derivative plot was smoothed using the moving window average method. Final melting temperatures are given as averages from the cooling curves of three independent measurements.

### CD spectroscopy

CD experiments were performed at 20 °C with a JASCO J‐810 spectropolarimeter equipped with a Peltier thermostat. Following stirring of the sample for 2 min to avoid concentration gradients in the cuvette, spectra were recorded from 210 to 350 nm with a bandwidth of 1 nm, a scanning speed of 50 nm/min and a response time of 4 s using a 1‐cm quartz cuvette. Five accumulations were recorded for each sample.

### NMR spectroscopy

NMR measurements were performed with a Bruker Avance Neo 600 MHz NMR spectrometer equipped with a ^1^H/^13^C/^15^N/^19^F quadruple resonance cryo‐probehead and z‐field gradients. Unless stated otherwise, experiments were performed at 30 °C. Data were processed in TopSpin 4.0.7 and analyzed using CcpNmr 2.4.2 software.[^28^, ^29^] For experimental details see the Supporting Information.

### Molecular dynamics simulation

Starting structures calculated with unmodified guanosines replacing 8‐bromo‐guanosine analogues were generated by simulated annealing in XPLOR‐NIH 3.0.3.[^30^, ^31^] Partial atomic charges for the modified 8‐bromoguanosine residue were calculated using the RED software with DFT approach.^[32]^ Refinement was performed using AMBER16 with the parmbsc force field and OL15 modifications.^[33]^ Adding potassium ions in explicit water, ten lowest‐energy structures were finally obtained after equilibration for 4 ns and short minimization *in vacuo*. RMSD calculations, alignments, and analyses of the calculated structures were performed with the VMD‐1.9.3 software.^[34]^ Three‐dimensional structural representations were prepared with Pymol‐1.8.4 [58].^[35]^ For details of the calculations see the Supporting Information.

### Isothermal titration calorimetry

ITC experiments were performed with a Microcal PEAQ ITC microcalorimeter (Malvern Instruments, United Kingdom) employing a reference power of 4 μcal s^−1^. Oligonucleotides and the PIQ ligand were each dissolved in 100 mM KCl, 20 mM potassium phosphate buffer, pH 7.0, supplemented with 5 % DMSO. The PIQ solution (400 μM) was titrated to 20 μM of oligonucleotide with a total of 2×26 injections of 1.5 μL each, an injection duration of 3 s, and a spacing between injections of 240 s. The first injection (0.4 μL) was rejected during the fitting process. All experiments were blank‐ and concentration‐corrected. For data analysis, the MicroCal PEAQ‐ITC analysis software was used.

## Accession Codes

Atomic coordinates and lists of chemical shifts for *L11AT‐B13* have been deposited (PDB ID 7O1H, BMRB ID 34615).

## Conflict of interest

The authors declare no conflict of interest.

## Supporting information

As a service to our authors and readers, this journal provides supporting information supplied by the authors. Such materials are peer reviewed and may be re‐organized for online delivery, but are not copy‐edited or typeset. Technical support issues arising from supporting information (other than missing files) should be addressed to the authors.

Supplementary
