# Supplementary material for: Expanding the Topological Landscape by a G‐Column Flip of a Parallel G‐Quadruplex
Source: Chemistry. 2021 Jun 4;27(40):10437–47. doi: 10.1002/chem.202101181 (PMC8361731; doi:10.1002/chem.202101181)
Supplement: Supplementary file 1 — Supplementary [file CHEM-27-10437-s001.pdf]

Chemistry–A European Journal

Supporting Information

Expanding the Topological Landscape by a G-Column Flip of a Parallel G-Quadruplex

Swantje Mohr, Jagannath Jana, Yoanes Maria Vianney, and Klaus Weisz*

METHODS

NMR spectroscopy. Experiments were generally performed at 303 K with proton chemical shifts referenced relative to the temperature-dependent water chemical shift at pH 7. Carbon chemical shifts were referenced to DSS using the indirect referencing method. For one-dimensional and two-dimensional NOESY spectra, an optimized WATERGATE with w5 element was used for solvent suppression. For HSQC experiments, the 3-9-19 solvent suppression scheme was employed for samples in 90% H_2O / 10% D_2O, using a spectral width of 6.7 kHz in the ^{13}C dimension. NOESY spectra were acquired with mixing times of 80, 150, or 300 ms in 90% H_2O / 10% D_2O. DQF-COSY spectra were recorded in 100% D_2O with solvent suppression through presaturation.

Molecular dynamics simulation. Initially, 100 starting structures of lowest energy were selected out of 400 structures generated by simulated annealing in XPLOR-NIH 3.0.3.[1,2] Distance restraints were set according to cross-peak intensities in 2D NOESY spectra. For exchangeable protons, peaks were categorized into very strong (2.9 ± 1.1 Å), strong (4.0 ± 1.2 Å), medium (5.0 ± 1.2 Å), and weak (6.0 ± 1.2 Å). For non-exchangeable protons, distances were set to 2.9 ± 1.1 Å for strong, 4.0 ± 1.5 Å for medium, 5.5 ± 1.5 Å for weak, 6.0 ± 1.5 Å for very weak, and 5.0 ± 2.0 Å for overlapping cross-peaks. Distance restraints for H-bonds were included for the tetrads and Watson-Crick base pairs. Glycosidic torsion angle restraints were set to *anti* (170-310°) for all residues except for Gs in the fourth G-tract which were restrained to *syn* (25-95°). Additional planarity restraints were only employed for the tetrads. Starting structures were calculated with unmodified guanosines replacing 8-bromo-guanosine analogs.

Partial atomic charges for the modified 8-bromoguanosine residue were calculated using the RED software with DFT approach.[3] Refinement was performed using AMBER16 with the parmbsc force field and OL15 modifications.[4] The 100 starting structures were subjected to simulated annealing *in vacuo* to yield 20 converged structures. Here, the same restraints were used as before with restraint energies set to 40 $kcal \cdot mol^{-1} \cdot Å^{-2}$ for NOE distance restraints, 50 $kcal \cdot mol^{-1} \cdot Å^{-2}$ for hydrogen bond distance restraints, 200 $kcal \cdot mol^{-1} \cdot rad^{-2}$ for dihedral angle restraints, and 30 $kcal \cdot mol^{-1} \cdot Å^{-2}$ for planarity restraints in tetrads and base pairs. The system was equilibrated at 300 K for 5 ps, followed by heating to 1000 K for 10 ps. This temperature was maintained for 30 ps, then the system was cooled to 100 K within 45 ps and to 0 K in 10 ps. Ten lowest-energy conformations were selected for further refinement in water.

Next, potassium ions were added to neutralize the system. Two of them were placed in the inner channel of the quadruplex core between tetrad layers. After placing each structure in a 10 Å octahedral box, TIP3P water was added. The simulation began with 500 steps of steepest descent followed by 500 steps of conjugate gradient minimization. Here, the DNA was fixed with a force constant of 25 kcal·mol^{-1}·Å^{-2}. In the following, the system was heated from 100 to 300 K during 10 ps in a NVT ensemble, continuing with a NPT ensemble equilibration at 1 atm with energy restraints decreasing from 5 to 0.5 kcal·mol^{-1}·Å^{-2}. Finally, a simulation for 4 ns at 1 atm and 300 K was calculated. The last 500 ps of the trajectory were averaged and shortly minimized *in vacuo*, resulting in 10 lowest-energy structures.

References

[1] C. D. Schwieters, J. J. Kuszewski, N. Tjandra, G. M. Clore, *J. Magn. Reson.* **2003**, *160*, 65–73.

[2] C. D. Schwieters, J. J. Kuszewski, G. M. Clore, *Prog. Nucl. Magn. Reson. Spectrosc.* **2006**, *48*, 47–62.

[3] E. Vanquelef, S. Simon, G. Marquant, E. Garcia, G. Klimerak, J. C. Delepine, P. Cieplak, F. Y. Dupradeau, *Nucleic Acids Res.* **2011**, *39*, W511–W517.

[4] M. Zgarbová, J. Šponer, M. Otyepka, T. E. Cheatham, R. Galindo-Murillo, P. Jurečka, *J. Chem. Theory Comput.* **2015**, *11*, 5723–5736.

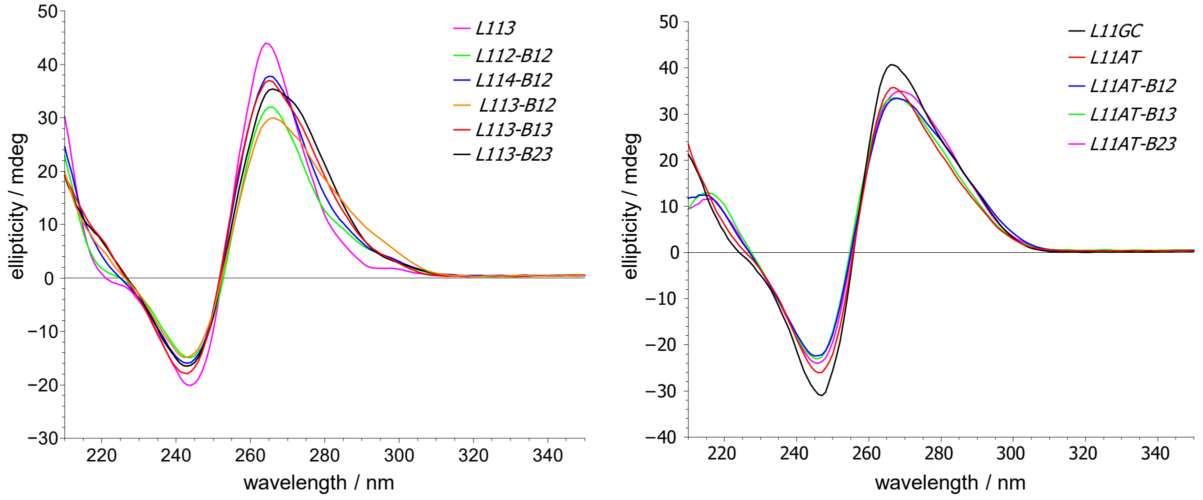

Figure S1. CD spectra of quadruplex-forming sequences without (left) and with a putative stem-loop structure (right) in 10 mM potassium phosphate buffer, pH 7.

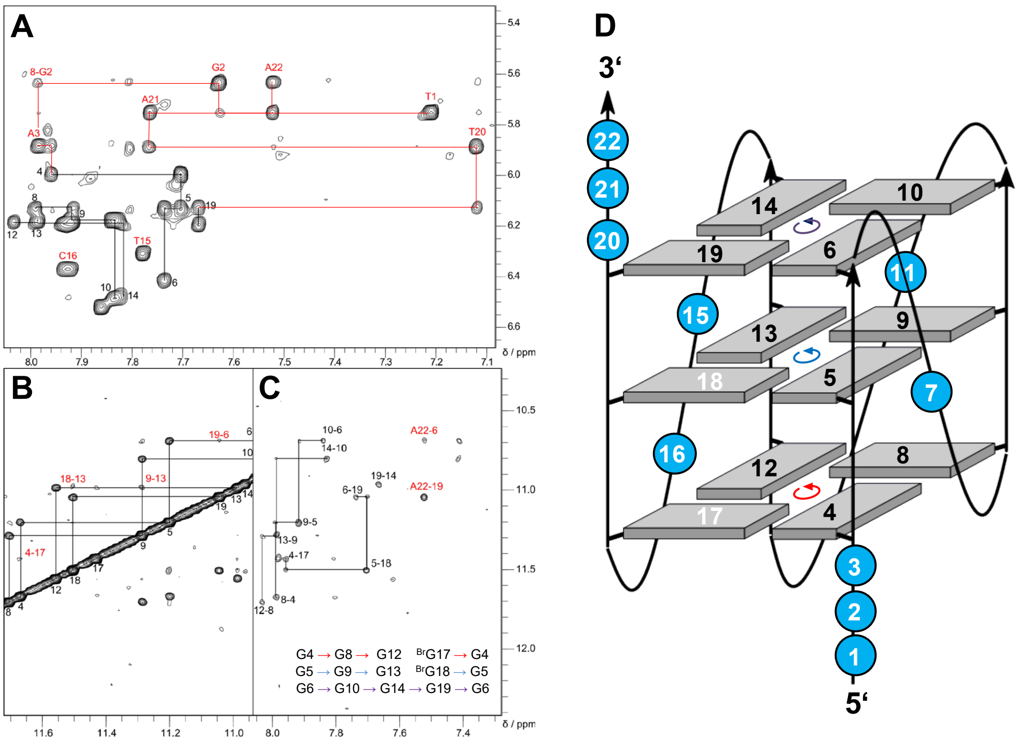

Figure S2. 2D NOE spectral regions of *L112-B12*. (A) H8/6(ω2)-H1'(ω1) cross-peaks with sequential contacts traced along the G-core (black) as well as along overhang residues (red). (B) Sequential H1-H1 cross-peaks and additional intra-tetrad contacts labeled in red. (C) Intra- and inter-tetrad H8(ω2)-H1(ω1) cross-peaks and additional contacts involving overhang residue A22 labeled in red. Tetrad polarities as determined from intra-tetrad NOE contacts are summarized at the bottom. (D) Topology of the *L112-B12* quadruplex with residue numbers and tetrad polarities indicated; ^{Br}G analogs are labeled with white numbers.

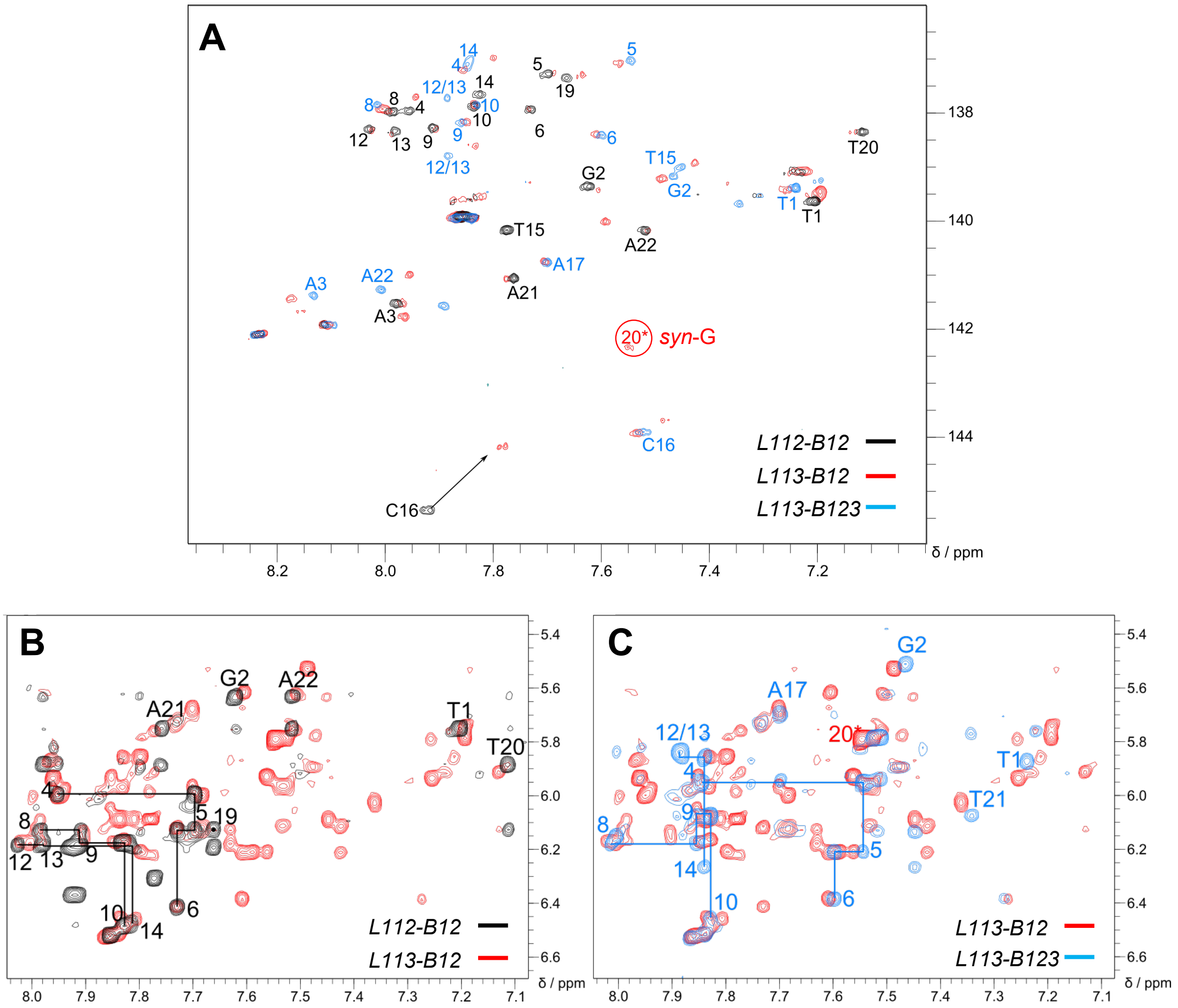

Figure S3. Spectral comparison through superimposed two-dimensional spectra of *L112-B12*, *L113-B12*, and *L113-B123*. (A) H6/8(ω2)-C6/8(ω1) correlations in ^{1}H-^{13}C HSQC spectra with corresponding assignments; correlations for *L113-B12* (red) match correlations of both *L112-B12* (black) and *L113-B123* (blue); a single *syn*-G (circled) can be assigned to G20 of the *L113-B12* major species. (B) H8/6(ω2)-H1'(ω1) cross-peaks of *L113-B12* (red) and *L112-B12* (black); sequential contacts are traced along the G-columns for *L112-B12*. (C) H8/6(ω2)-H1'(ω1) cross-peaks of *L113-B12* (red) and *L113-B123* (blue); sequential contacts are traced along the G-columns for *L113-B123*.

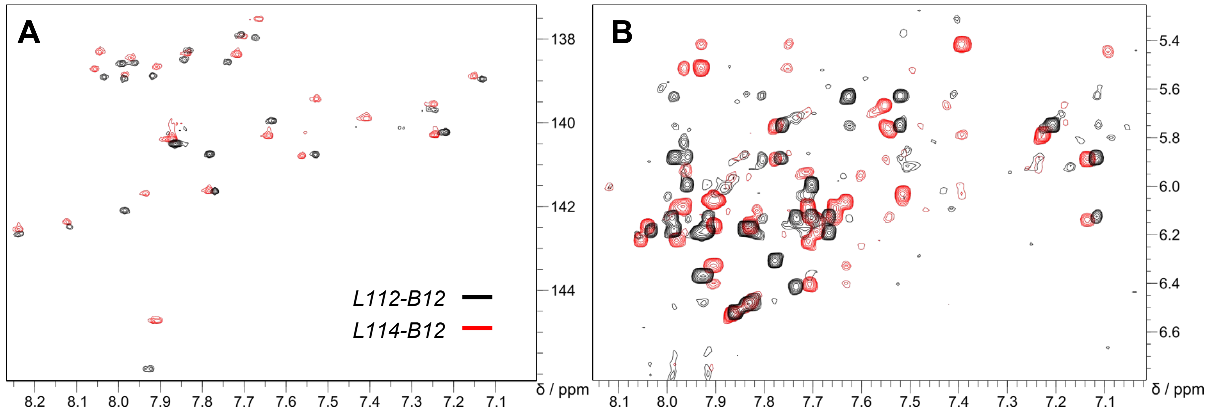

Figure S4. Spectral comparison through superimposed two-dimensional spectra of *L114-B12* (red) and *L112-B12* (black). (A) H6/8(ω_2)-C6/8(ω_1) correlations in ^{1}H-^{13}C HSQC spectra and (B) H8/6(ω_2)-H1'(ω_1) correlations in 2D NOESY spectra.

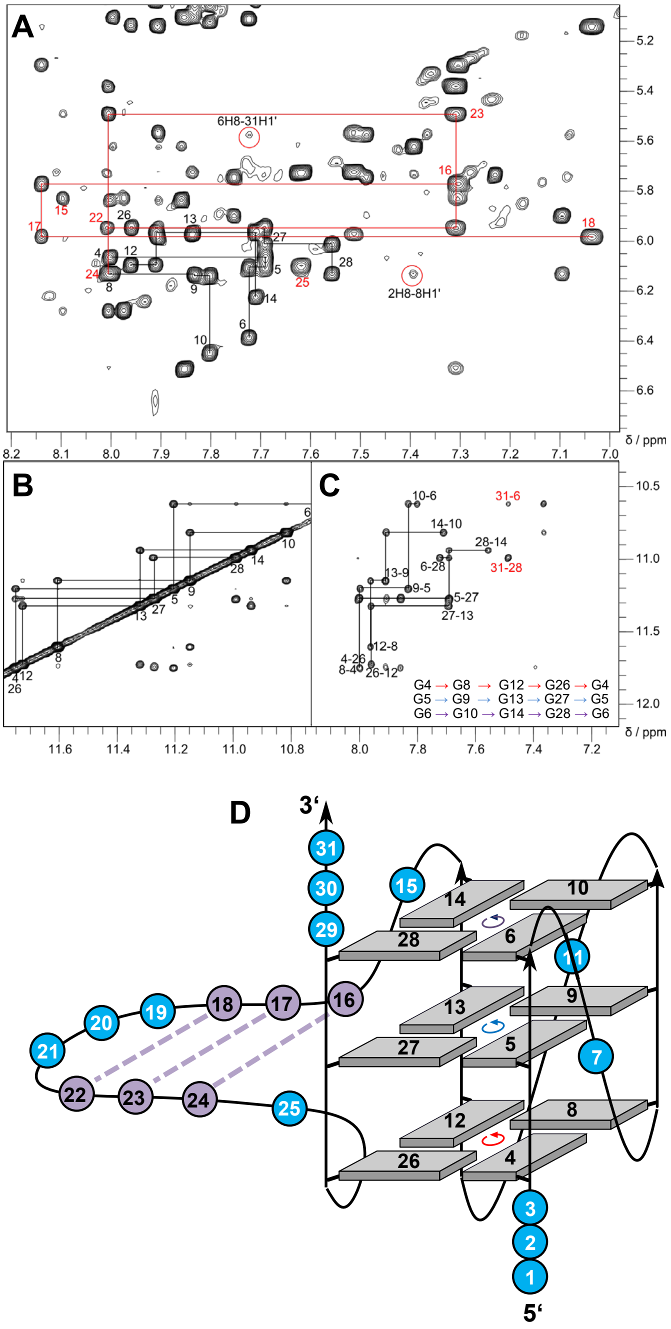

Figure S5. 2D NOE spectral regions of *L11GC*. (A) H8/6(ω2)-H1'(ω1) cross-peaks with sequential contacts traced along the G-core (black) and the duplex stem (red); NOE contacts between overhang residues and outer G-tetrads are marked by red circles. (B) Sequential H1-H1 cross-peaks along the G-columns. (C) Intra- and inter-tetrad H8(ω2)-H1(ω1) cross-peaks and additional contacts involving overhang residue A31 labeled in red. Tetrad polarities as determined from intra-tetrad NOE contacts are summarized at the bottom. (D) Topology of the *L11GC* quadruplex with residue numbers and tetrad polarities indicated; GC Watson-Crick base pairs within the 11-nt propeller loop are indicated by broken violet lines.

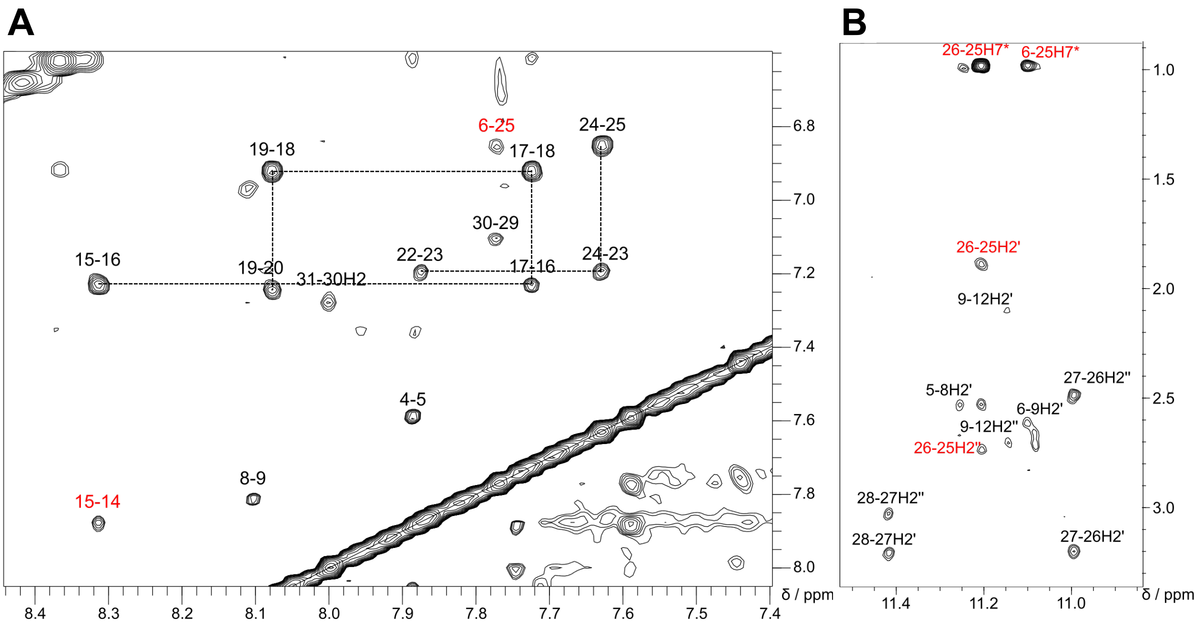

Figure S6. (A) H8/6-H8/6 and (B) H1(ω2)-H2'/H2''(ω1) 2D NOE spectral region of *L11AT-B13*; contacts at the quadruplex-duplex interface are labeled in red. Contacts along the duplex stem are traced by horizontal and vertical lines in (A).

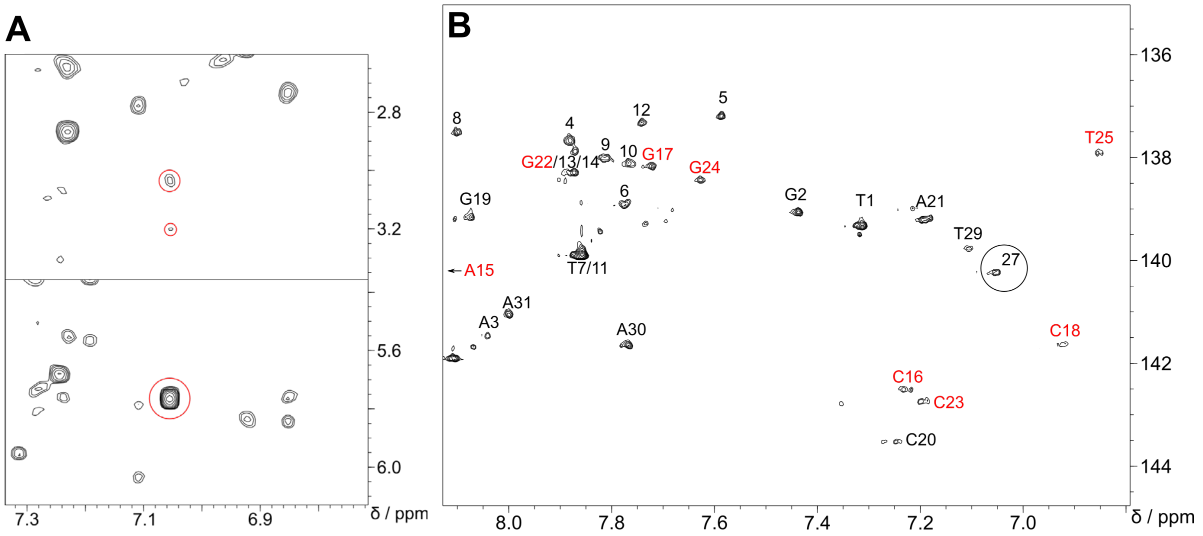

Figure S7. (A) 2D NOESY spectrum of *L11AT-B13;* H8/H6(ω_2)-H2'/H2"(ω_1) (top) and H8/H6(ω_2)-H1'(ω_1) NOE contacts (bottom) with weak and strong intra-residual cross-peaks marked for G27 (80 ms mixing time). (B) H6/8-C6/8 region of a ^{1}H-^{13}C HSQC spectrum of *L11AT-B13*; cross-peaks from Watson-Crick base pairs are marked in red; *syn*-G27 is circled.

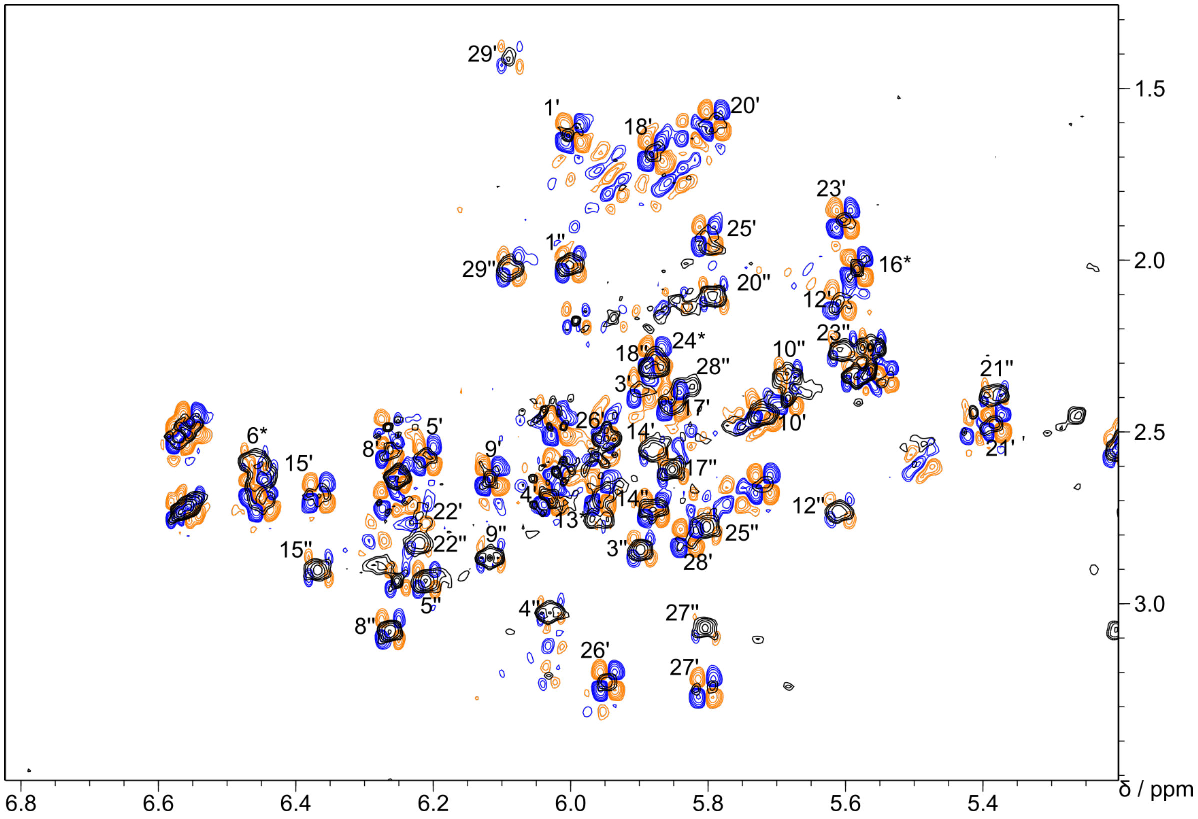

Figure S8. Superimposed H1'-H2'/H2" spectral region of NOESY (80 ms mixing time, black) and DQF-COSY spectra (blue/orange) for *L11AT-B13*; H1'-H2' and H1'-H2" contacts are labeled with residue number; correlations of stereochemically ambiguous resonances are marked with an asterisk.

Table S1. ^1H and ^{13}C chemical shifts δ (ppm) of *L112-B12*.[a]

residue	H8/H6	C8/C6	H1'	H2'/H2"[b]	H1	H5/H2/Me
T1	7.21	139.73	5.75	2.04	n.d.	n.d.
G2	7.63	139.45	5.64	2.38 / 2.45	n.d.	-
A3	7.98	141.63	5.88	2.59	-	n.d.
G4	7.96	138.07	6.00	2.72 / 2.96	11.67	-
G5	7.70	137.41	6.13	n.d.	11.20	-
G6	7.73	138.03	6.42	2.61 / 2.75	10.69	-
T7	7.86	139.94	6.53	n.d.	n.d.	1.98
G8	7.99	138.08	6.13	2.46 / 2.94	11.71	-
G9	7.92	138.37	6.18	2.65 / 2.91	11.29	-
G10	7.84	137.99	6.49	2.64 / 2.77	10.80	-
T11	7.86	139.94	6.53	n.d.	n.d.	1.98
G12	8.03	138.41	6.19	2.46 / 2.97	11.56	-
G13	7.99	138.44	n.d.	n.d.	10.99	-
G14	7.83	137.77	n.d.	n.d.	10.96	-
T15	7.78	140.26	6.31	2.34 / 2.51	n.d.	1.97
C16	7.93	145.46	6.37	2.52 / 2.68	-	n.d.
^{Br}G17	-	-	n.d.	n.d.	11.43	-
^{Br}G18	-	-	6.20	2.63 / 3.14	11.50	-
G19	7.67	137.46	6.13	2.56 / 2.79	11.04	-
T20	7.12	138.45	5.89	1.90 / 2.37	n.d.	1.52
A21	7.76	141.17	5.75	2.00 / 2.46	-	n.d.
A22	7.52	140.28	5.63	2.19	-	n.d.

[a] At 303 K in 10 mM potassium phosphate buffer, pH 7.
[b] No stereochemical assignment for H2'/H2" protons.

Table S2. ^{1}H and ^{13}C chemical shifts δ (ppm) of *L113-B123*.[a]

residue	H8/H6	C8/C6	H1’	H2’/H2”[b]	H1	H5/H2/Me
T1	7.24	139.41	5.88	n.d.	n.d.	n.d.
G2	7.47	139.17	5.51	n.d.	n.d.	-
A3	8.14	141.40	5.96	n.d.	.	7.76
G4	7.85	137.12	5.96	2.66 / 2.92	11.32	-
G5	7.55	137.03	6.21	2.54 / 2.89	10.94	-
G6	7.60	138.42	6.39	2.66	10.97	-
T7	7.87	139.96	6.52	2.46 / 2.70	n.d.	1.98
G8	8.02	137.87	6.18	2.45 / 3.01	11.53	-
G9	7.86	138.18	6.08	2.67 / 2.85	11.04	-
G10	7.84	137.86	6.46	2.57 / 2.67	11.15	-
T11	7.87	139.96	6.52	2.46 / 2.70	n.d.	1.98
G12	7.89	n.d.	5.84	n.d.	10.99	-
G13	7.89	n.d.	5.86	2.66	11.09	-
G14	7.84	137.04	6.27	2.65 / 2.78	11.16	-
T15	7.45	139.01	6.14	2.20 / 2.42	n.d.	1.71
C16	7.52	n.d.	5.94	1.98 / 2.28	-	5.78
A17	7.70	140.78	5.70	1.80 / 2.42	-	7.29
^{Br}G18	-	-	n.d.	n.d.	11.21	-
^{Br}G19	-	-	n.d.	n.d.	11.49	-
^{Br}G20	-	-	n.d.	n.d.	11.45	-
T21	7.34	141.89	6.08	n.d.	n.d.	1.88
A22	8.01	141.28	6.16	n.d.	-	n.d.
A23	n.d.	n.d.	n.d.	n.d.	-	7.44

[a] At 303 K in 10 mM potassium phosphate buffer, pH 7.
[b] No stereochemical assignment for H2’/H2” protons.

Table S3. [1]H and ^{13}C chemical shifts δ (ppm) of *L11GC*.[a]

residue	H8/H6	C8/C6	H1’	H2’/H2”[b]	H1	H5/H2/Me
T1	7.23	139.41	5.74	n.d.	n.d.	1.62
G2	7.39	139.74	5.62	2.33 / 2.83	n.d.	-
A3	7.86	n.d.	5.84	n.d.	-	n.d.
G4	8.00	n.d.	6.07	3.03	11.75	-
G5	7.69	137.40	6.10	2.88	11.21	-
G6	7.72	137.85	6.39	2.59 / 2.74	10.62	-
T7	7.85	139.91	6.51	2.45 / 2.67	n.d.	1.97
G8	8.00	137.75	6.13	2.44 / 2.93	11.60	-
G9	7.83	138.14	6.14	2.60 / 2.87	11.15	-
G10	7.80	137.78	6.45	2.61 / 2.75	10.82	-
T11	7.85	139.91	6.51	2.45 / 2.67	n.d.	1.97
G12	7.96	n.d.	6.09	n.d.	11.73	-
G13	7.91	n.d.	5.97	n.d.	11.32	-
G14	7.71	137.54	6.23	n.d.	10.94	-
G15	7.84	138.97	n.d.	n.d.	n.d.	-
C16	7.31	143.63	5.77	2.48 / 2.69	-	5.30
G17	8.14	139.31	5.98	2.38 / 2.70	n.d.	-
C18	7.04	141.77	5.99	n.d.	-	5.14
G19	7.91	n.d.	5.57	n.d.	n.d.	-
C20	7.51	143.38	5.97	n.d.	-	n.d.
A21	7.84	n.d.	n.d.	n.d.	-	n.d.
G22	8.01	n.d.	5.95	1.65 / 2.22	n.d.	-
C23	7.31	142.63	5.49	2.54 / 2.58	-	5.38
G24	8.01	138.76	6.13	n.d.	n.d.	-
C25	7.62	144.00	6.10	2.36 / 2.46	-	5.73
G26	7.96	n.d.	5.94	2.53 / 2.79	11.75	-
G27	7.69	137.40	6.02	2.62 / 2.68	11.28	-
G28	7.56	137.06	6.13	2.50 / 2.78	10.99	-
T29	7.10	138.25	5.90	n.d.	n.d.	1.43
A30	7.75	141.07	5.75	n.d.	-	7.08
A31	7.49	140.19	5.58	n.d.	-	7.37 / 153.9

[a] At 303 K in 10 mM potassium phosphate buffer, pH 7.
[b] No stereochemical assignment for H2’/H2” protons.

Table S4. ^{1}H and ^{13}C chemical shifts δ (ppm) of *L11AT-B13*.[a]

residue	H8/H6	C8/C6	H1'	H2'/H2"[b]	H3'	H1	H5/H2/C2/Me
T1	7.31	139.34	5.96	1.60 / 1.99	4.39	n.d.	1.74
G2	7.44	139.07	5.53	2.24 / 2.30	4.67	n.d.	-
A3	8.04	141.47	5.87	2.36 / 2.81	4.73	-	7.74 / 154.9
G4	7.88	137.68	5.99	2.67 / 3.00	4.90	11.01	-
G5	7.59	137.20	6.18	2.54 / 2.91	4.80	11.26	-
G6	7.77	138.92	6.42	2.67*	4.65	11.10	-
T7	7.86	139.91	6.52	2.46 / 2.69	n.d.	n.d.	1.99
G8	8.10	137.50	6.23	2.53 / 3.05	5.17	11.52	-
G9	7.81	138.03	6.08	2.61 / 2.84	5.06	11.15	-
G10	7.77	138.11	5.66	2.37 / 2.33	4.65	11.09	-
T11	7.86	139.91	6.52	2.46 / 2.69	n.d.	n.d.	1.99
G12	7.74	137.34	5.59	2.11 / 2.71	5.12	11.05	-
G13	7.89	n.d.	5.93	2.66* / 2.72*	5.07	11.61	-
G14	7.88	n.d.	5.84	2.70 / 2.70	5.07	10.68	-
A15	8.31	141.25	6.33	2.65 / 2.87	5.11	-	7.76 / 155.1
C16	7.23	142.51	5.55	2.00* / 2.31*	4.84	-	5.22
G17	7.72	138.18	5.82	2.39 / 2.58	4.90	12.75	-
C18	6.92	141.65	5.84	1.66 / 2.28	4.74	-	5.03
G19	8.08	139.14	5.68	2.63 / 2.43	4.83	n.d.	-
C20	7.24	143.52	5.76	1.58 / 2.08	4.37	-	5.23
A21	7.19	139.20	5.35	2.45 / 2.37	n.d	-	n.d.
G22	7.88	n.d.	6.18	2.72 / 2.79	4.77	12.81	-
C23	7.19	142.75	5.57	1.85 / 2.23	4.69	-	5.16
G24	7.63	138.45	5.84	2.25* / 2.52*	4.80	12.76	-
T25	6.85	137.92	5.76	1.90 / 2.74	4.73	13.45	1.00
^{Br}G26	-	-	5.91	3.21 / 2.50	n.d.	11.21	-
G27	7.05	140.25	5.77	3.21 / 3.04	4.88	11.00	-
^{Br}G28	-	-	5.79	2.78 / 2.34	4.89	11.42	-
T29	7.11	139.78	6.04	1.41 / 2.01	4.69	n.d.	1.85
A30	7.77	141.63	5.66	3.24 / 3.24	4.65	-	7.28 / 154.5
A31	8.00	141.06	6.20	2.60 / 2.89	4.60	-	n.d.

[a] At 303 K in 10 mM potassium phosphate buffer, pH 7.
[b] Stereochemically ambiguous H2'/H2" resonances are marked with an asterisk.

Table S5. NMR restraints and structural statistics for the structure calculations of *L11AT-B13*.

NMR restraints	
NOE-based distance restraints:	
intra-residual	97
inter-residual	153
exchangeable	62
other restraints:	
hydrogen bonds	70
dihedral angles	31
planarity	3
structural statistics	
pairwise heavy atom RMSD value (Å):	
all residues	2.5 ± 0.4
G-tetrad core	0.5 ± 0.1
duplex stem-loop	2.5 ± 0.9
NOE violations (Å):	
maximum violation	0.30
mean NOE violation	0.003 ± 0.001
deviations from idealized geometry:	
bonds (Å)	0.01 ± 0.0001
angles (degree)	2.25 ± 0.02
